# An interplay of resource availability, population size and mutation rate potentiates the evolution of metabolic signaling

**DOI:** 10.1186/s12862-021-01782-0

**Published:** 2021-04-07

**Authors:** Bhaskar Kumawat, Ramray Bhat

**Affiliations:** Department of Molecular Reproduction, Development and Genetics, Indian Institute of Science, Bangalore, 560012 India

**Keywords:** Evolution, Signaling, Artificial life, Tumorigenesis, Development

## Abstract

**Background:**

Asexually reproducing populations of single cells evolve through mutation, natural selection, and genetic drift. Environmental conditions in which the evolution takes place define the emergent fitness landscapes. In this work, we used Avida—a digital evolution framework—to uncover a hitherto unexplored interaction between mutation rates, population size, and the relative abundance of metabolizable resources, and its effect on evolutionary outcomes in small populations of digital organisms.

**Results:**

Over each simulation, the population evolved to one of several states, each associated with a single dominant phenotype with its associated fitness and genotype. For a low mutation rate, acquisition of fitness by organisms was accompanied with, and dependent on, an increase in rate of genomic replication. At an increased mutation rate, phenotypes with high fitness values were similarly achieved through enhanced genome replication rates. In addition, we also observed the frequent emergence of suboptimal fitness phenotype, wherein neighboring organisms signaled to each other information relevant to performing metabolic tasks. This metabolic signaling was vital to fitness acquisition and was correlated with greater genotypic and phenotypic heterogeneity in the population. The frequency of appearance of signaling populations increased with population size and with resource abundance.

**Conclusions:**

Our results reveal a minimal set of environment–genotype interactions that lead to the emergence of metabolic signaling within evolving populations.

**Supplementary Information:**

The online version contains supplementary material available at 10.1186/s12862-021-01782-0.

## Background

Populations of mitotically dividing cells and unicellular organisms evolve under the complex regulation of their environments. This regulation can be exerted through variations in abundance of metabolizable resources available to the population. Elegant experiments using unicellular budding yeast grown in low density sucrose-containing environments show that multicellularity can evolve under resource-poor conditions with cooperation between incompletely separated cell populations [1]. Extending such observations, resource availability is theorized to have played a key role in determining the evolution of developmental mechanisms with resource-rich environments considered more ideal for the evolutionary stabilization of uniclonal, rather than polyclonal populations [2].

Upper bounds on population size have also been suggested to play a contextual role in evolution: small populations are more susceptible to random genetic drift with frequent fixation of deleterious mutations. Above a certain threshold of population size, additional beneficial mutations could rescue such populations [3]. On the other hand, LaBar and Adami [4] show that smaller populations can evolve robustness against genetic drift by adapting to lower but more stable peaks on the fitness landscape—i.e. those with lesser probability of small-effect deleterious mutations. Another exploration of a similar problem under an evolutionary game theory framework suggests that small population sizes, consisting of participants that can memorize strategies and interaction outcomes, evolve to cooperate easier than memory-less populations by storing these outcomes and using them to guide future interactions [5].

The role of mutation rates in the evolution of asexual populations has been theoretically explored by several works [6–8]. Individual mutations may have a deleterious, neutral or beneficial effect on the fitness of the populations within which they arise; fitness-enhancing mutations constitute only a small proportion within this possible set [9]. However, the rate of adaptation has been shown to increase under high mutation rates [10], but this is not without a limit: very high mutation rates can lead to a catastrophic decline in fitness (mutational meltdown) [11]. Increased mutation rates within heterogeneous populations result in the evolution of populations to lower fitness values while attaining higher robustness: a hypothesis known as the “survival of the flattest” [12, 13]. Furthermore, in experimental models, increased mutagenesis has been shown to be a genotypic response to environmental stresses [14–16] and can in turn increase evolvability [17].

Organisms inhabiting the natural world evolve within their diverse and often variable environments, with all the above factors simultaneously constraining and biasing their phylogeny. In this article, we examine the interplay between the three factors—mutation rate, resource availability and maximum population size and ask whether these factors amplify or offset each other’s effects in determining the likelihood of populations evolving specific strategies. This examination is relevant to the evolution of discrete populations of cancer cells such as within spheroids and multicellular circulating tumor clusters that inhabit, and transit through, microenvironments with varying resource abundances. Such populations have been shown to comprise of cells with heterogeneity in genotype and phenotype, although the cues that engender the emergence of heterogeneity are unclear [18–20]. We use the Avida artificial life platform, wherein mutating, reproducing, and resource-metabolizing digital organisms are allowed to evolve under distinct values of these variables. This platform has been used by a large number of groups to address important questions pertaining to evolutionary dynamics of asexually reproducing organisms. For example, Wilke and coworkers have shown that high mutation rates bias the emergence of low fitness genotypes that lie in a region of relatively high stability on the fitness landscape rather than more fragile, high-fitness genotypes [13]. Labar and Adami [4] have highlighted the appearance of drift robustness in small populations of digital organisms [4]. Goldsby and coworkers show that an increase in mutation rates induced by metabolic processes (like DNA damage and repair due to reactive oxygen species) can induce differentiation of cell clusters into soma and germline—with the somatic cells performing the majority of the metabolic processes [21]. In this paper, using this framework, we analyze the constitution and frequency of predominant genotypes emerging from the evolutionary runs for ancestral organisms that begin with a basic reproduction machinery and the ability to perform a simple metabolic task. Our work sheds light on the necessary conditions for the emergence of distinct fitness landscapes for small populations in different environments.

## Methods

### Avida computational model

The Avida digital evolution platform is a computational framework that allows simulation of self-replicating organismal populations in a fixed-size, lattice-based virtual world [22]. The organisms in Avida consist of a genome sequence composed of a series of genotypic instructions (similar to genes on chromosomes). In our simulations, the sequence size is limited to 120 instruction-sites (analogous to loci) and each instruction-site can contain one of 32 instructions chosen from an instruction-set. The instruction-set is Turing-complete and allows these organisms to copy the genome, replicate by division, perform logical/mathematical operations, control the flow of execution (using jumps and loops), perform input/output to interact with the environment or send information to neighbors (refer to Additional file 1: S2 for a complete list of Avida instructions). The environment provides 5-bit numbers as metabolic substrates that the organisms can read and manipulate in order to generate a resultant output. If the genome is able to output a specific calculation on the inputs, the organisms take up a certain fraction of an external *resource* and are rewarded a corresponding *merit*, a quantity that essentially determines the speed with which an organism genome is executed. This is similar to how real cells can convert simple molecules into more readily utilizable products like ATP or NADPH in order to increase their replication rate, which in turn increases fitness. These molecules also act as potent signaling molecules between cells [23, 24]. As an example, if the environment rewards performance of a logical AND task, an organism which can take two environmental inputs A and B (generated randomly when organisms execute an input instruction) and can output “A AND B” gets a merit reward. In turn, genomes that execute faster, copy faster and therefore give rise to more offspring per unit time. These resources are implemented as a global chemostat that maintains a certain level of resources in each lattice-site in the world (each containing one organism) using fixed inflow and outflow rates. All offspring also inherit a basal merit from their parents and thus do not necessarily require the presence of metabolic tasks to survive if they can reproduce before the internal merit reserve is depleted.

To accomplish reproduction, the organisms also need to have a working copying-and-division mechanism that is implemented in the ancestral seed genome as a *copy-loop*. This ancestral copy-loop has two main functions—(a) To copy the parent genome instruction-by-instruction until the entire genome has been copied, and (b) to divide the offspring after the copying is complete and place this offspring in a new neighboring site in the world. The organisms have a faced direction in the world and can *rotate* in order to choose either the receiving neighbor (for metabolic signals) or to choose the direction in which to place an offspring. If there is already an organism in the site, it is replaced by the newly created offspring. The world is limited in size and thus cannot accommodate more than a fixed number of organisms.

Apart from sending generated task results to the environment for verification and reward, the organisms are also able to send the numbers stored in their memory to their eight nearest neighbors by executing a set of instructions. This is again performed in a directional fashion, with the messages being sent to the organism being faced. These *messaging* instructions can either send intermediary metabolic information (the incomplete result of a task) or allow inter-cellular regulation by acting as a signal for other genomic instructions. For example, the OR task requires a smaller number of genomic instructions to execute than NOR and hence is more likely to appear randomly through mutations. An OR-performing organism can share the result of this task with a neighbor, which can then perform a NOT operation on it to output NOR—a task with a much higher utility than NOT and OR combined. The cost of computation is thus shared between the two organisms. In return, the neighbor can return this information (the NOR output given two inputs) to the initial sender and allow it to accrue a benefit. Note that there is no direct cost to messaging, except it decreases the available genomic space to evolve other metabolic mechanisms. A natural example of this can be seen in *Dictyostelium discoideum*, where signaling between neighboring cells allows the cells to group together, undergo differentiation, and survive starvation [25, 26].

The Avida organisms are thus free to innovate in three major areas—metabolism, reproduction, and information sharing (or signaling). The set of instructions that are relevant in our study and allow the organisms to innovate these mechanisms is given in Table 1. There are a total of nine tasks available in the environment, each with a corresponding resource. The list of these tasks and their relative payoffs to the merit (replication rate) are given in Table 2.

**Table 1:** Major instructions of interest in Avida and their functions

Instruction	Function
Flow-control: Control the flow during genome execution which is otherwise linear	
if-label (f)	Executes the next instruction in the genome sequence if the flow head is at a specified label. In the ancestral copy-loop, this instruction makes sure that the division instruction is executed only when the entire genome has been copied.
Reproduction (Biological): Assist in copying of genome sequence during replication	
h-copy (v)	Copies a single instruction from parent genome to offspring genome
h-divide (x)	Separates offspring genome from parent and places the offspring organism in the cell faced by parent
h-search (z)	Finds a specified label in the genome and moves a specified head to this label. In the copy-loop, this instruction ensures the flow head returns to the start of the loop on reaching the end of the genome.
Messaging: Allow organisms to send and receive messages	
send-msg (B)	Send a message (containing numbers stored in two of the registers) to the neighbor in the direction the organism currently faces.
retrieve-msg (C)	Retrieves messages into specified registers from the messaging buffer
bcast1 (D)	Sends a message to all the eight nearest neighbors
Rotation: Allow organisms to change the direction they are facing	
rotate-left-one (E)	Rotates the organism by a unit in the anticlockwise direction
rotate-right-one (F)	Rotates the organism by a unit in the clockwise direction

Note that this list consists of only nine of the most important instructions out of the 32 instructions in our simulations. For the complete list of instructions and their functions please refer to additional information. Some of the terminology used in this table requires an understanding of the Avida organism life-cycle (underlined) and is presented concisely in the additional information

**Table 2:** Metabolic tasks in the environment and their merit payoffs. Adapted from [52]

Task	Operation (Required output given two binary numbers A and B)	Merit reward
NOT	~A	1
NAND	~ (A ∧ B)	1
AND	A ∧ B	2
ORN	A ∨ ~B, ~A ∨ B	2
OR	A ∨ B	4
ANDN	A ∧ ~B, ~A ∧ B	4
NOR	~ (A ∨ B)	8
XOR	(A ∧ ~B) ∨ (~ A ∧ B)	8
EQU	(A ∧ B) ∨ (~ A ∧ ~B)	16

A particular task is performed when the organism outputs the operation corresponding to it after taking in two random inputs—A and B—from the environment. The merit reward denotes the relative magnitude of the addition to genome replication speed when a given task is performed. The symbols used in the table are: “~” denotes logical negation, “∨” denotes the logical-or function, and “∧” the denotes logical-and function. Some examples for these operations are given in Additional file 1: Table S1

### Experimental setup

**Fig. 1:**
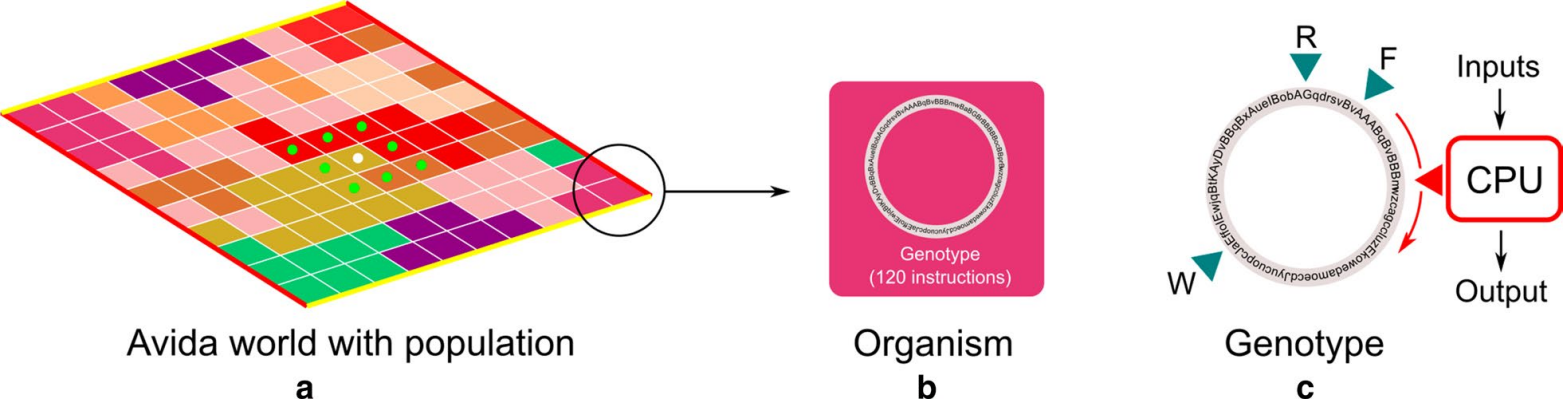
A schematic of the Avida world and its constituent organisms. **a** The Avida world consists of a 2D grid of lattice sites where organisms can reside. Each organism (e.g. the white dot) has eight nearest neighbors (and corresponding lattice sites, denoted by green dots) to whom it can send messages or replace with its offspring. The world is periodic, with the horizontal and vertical boundaries coinciding with their opposites (red boundary on red, yellow boundary on yellow). **b** Each organism consists of a genome containing 120 genomic instructions which are read and executed by a “virtual CPU”. **c** This virtual CPU reads the genome instruction by instruction, gets inputs from the environment and processes them using the genomic instructions to generate an output. It can also read and write instructions using the read (R) and write (W) heads in order to copy the genome before division

For all the experiments presented here, a single ancestral organism is released in an Avida world with periodic boundaries (lattice sites on the leftmost boundary are neighbors of lattice sites on the rightmost boundary, and similarly for the top and bottom boundaries) and allowed to reproduce to give rise to a population that has evolved over 100,000 updates (update being the basic unit of time in Avida; On average, around 30 genomic instructions are executed in a single update for each organism in the population). Simulations are run with two values of the mutation rate, at different levels of resource availability, and population size conditions. The ancestral genotype consists of a simple copy-loop with a primitive task definition that allows it to perform the simplest task in the environment. It also has a single rotation instruction, which allows the organism to change the direction it faces in the world and thus can facilitate messaging and/or dispersal of offspring after division (Fig. 1).

Mutation rates in Avida are denoted by probabilities of mutation of an instruction in a particular site. As each instruction has a larger functional role in the genome than a single base-pair in DNA genomes, this can be considered as a per-locus mutation rate. Under the high mutation rate, there is a 7.5 × 10^− 3^ probability of a genomic instruction being substituted with another on divide. Under the low mutation rate, substitutions are made with a probability of 7.5 × 10^− 4^, an order of magnitude lower. The sequence length is restricted to 120 and mutations events are limited to substitutions to conserve this length. In our simulations there are a total of 32 such instructions that can be substituted in any genomic site during mutation. Other than nop-X (a null instruction), each instruction has a direct functional role. For example, substitution of a “nop-X” with a “send-msg” instruction will pass a message to the currently faced neighbor.

Each task in the Avida world has an associated resource that can be taken up by an organism and an increase in replication rate (merit) is awarded proportional to these resources. Resources are modelled to have an inflow and outflow rate for each site in the world but can also diffuse laterally. These inflow and outflow rates maintain a steady concentration of each resource like in a chemostat. Resource heterogeneity in the world can arise if a certain localized cluster of cells is highly metabolically active. However, the small size of the world and high rate of diffusion between sites ensures this heterogeneity does not persist over evolutionary time. The steady-state resource availability is varied between multiple values, each an order of magnitude higher than the previous one. The lowest of these is at an absolute value of 0.1 (per-resource) and is chosen such that there are no secondary effects that restrict proper mutation and selection over the duration of the experiments (such as widespread extinctions). These values go all the way up to a value of 10,000 resource per lattice site. The world size (or maximum population size) is varied from 50 to 500 lattice sites (each lattice site can accommodate one organism) in keeping with our motivation to uncover emergence fitness landscapes of evolving discrete populations such as cancer spheroids and clusters. Each simulation condition is repeated for 100 replicate populations seeded with the same ancestral genome.

### Fitness distribution analysis for major innovations

Avida has an internal measure of fitness that is calculated by taking a ratio of the metabolic rate (i.e. merit) to the gestation time of an organism (time required for replication from the start of genome execution). However, both these values are calculated with the organism in isolation and thus the definition does not account for benefits to fitness due to intercellular signaling. To remedy this, we use a slightly modified metric that places 200 copies of the organism in a world, lets the population stabilize for a hundred updates and then calculates the average number of births per update for the next 400 updates. The mutation rate is set to zero during the entire process and thus prevents the sequences from evolving. This metric captures signaling between organisms with the same genotypes and does not account for cooperation between widely differing sequences. Our population sizes are kept small in order to obtain unimodal populations containing single dominant genotypes. We hence assume that the benefits to fitness due to signaling are obtained by sharing between homogeneous genotypes.

**Fig. 2:**
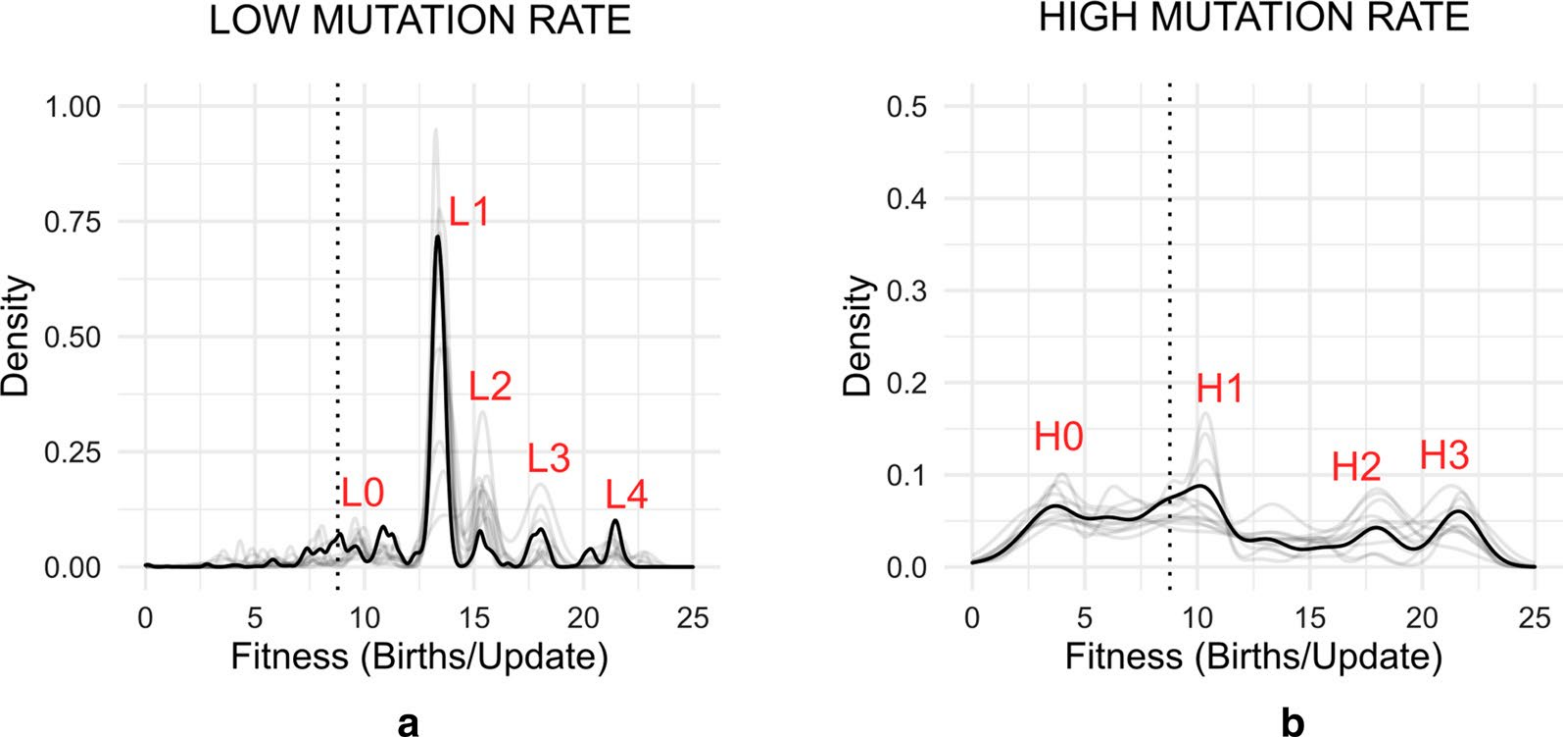
Fitness distribution of genotypes obtained from simulation at low and high mutation rates of a population of an ancestral organism (Dark line: resource levels of 10 k and population size of 500; Light lines: all other combinations of these variables, see Additional file 1: Fig. S4). **a** At low mutation rate, five major peaks (labeled L0–4) are obtained in the distribution. **b** At a high mutation rate, the distribution becomes more heterogeneous and four major peaks (H0 to H3) are observed. The dotted vertical line for both spectra represents the fitness of the ancestral organism. Y axis represents density (calculated from a kernel density estimate with area under the curve normalized to 1) and X axis represents fitness (defined as the number of births/updates)

Each value of the mutation rate leads to a distinct set of major peaks that appear on the fitness distribution (as discussed in  "Results"). To generate this fitness distribution, we collect the genotypes from all the treatments and replicates under a mutation rate and plot the kernel density estimate (KDE) for the fitness values. The high-density peaks that appear are taken as representatives of different mechanisms that emerge with a high probability. As discussed later, resource levels and maximum population size do not alter the position of these peaks but rather determine the probability of finding a genotype within these states (Fig. 2).

The genotypes from each peak are sampled by considering a narrow region around the peak maxima (within two times the bandwidth of the kernel density estimate). These sampled genotypes are then analyzed for common features to deconvolute the underlying mechanisms that give rise to the corresponding peaks.

### Marginal utility

We define a metric called the marginal utility of a process—defined as the fractional loss in fitness when the process is perturbed. This metric is calculated as follows.

$$\text{Marginal utility}\;=(\text{Fitness}\,-\,\text{Fitness without instructions that allow the process})/\text{Fitness}$$

Fitness without the process instructions is calculated by substituting all the relevant instructions in the genotype sequence with a null instruction (nop-X) and measuring the fitness of the resultant sequence.

### Isolation of signaling positive populations

The marginal utility of signaling for a genotype is calculated as the fractional loss in fitness when signaling/messaging instructions are removed from the genome. Marginal utility of signaling for all genotypes obtained in all the runs classified under two major peaks on a density distribution (Additional file 1: Fig. S6). The first peak was centered at zero and did not acquire fitness through signaling. The second peak showed a positive shift and consisted of genotypes that acquired a part of their fitness through signaling instructions. The midpoint of these peaks was chosen as a threshold to identify evolved populations as signaling or non-signaling populations. Populations with median marginal utility of messaging above this threshold were labelled as messaging positive populations.

### Genotypic heterogeneity of the population

Each genome sequence is restricted to a length of 120 sites (each containing an instruction) and this number is maintained through the mutation process by disallowing additions and deletions. To estimate the genetic heterogeneity in a population, we calculate the per-site entropy for the set of genomes in each of these populations and sum these values for all 120 genomic sites. For each position $i$ in the genotype sequence, the per-site entropy is given by,

$${s_i}= -\sum _{\text{instruction $j$}}{p}_{j}^{i} \ln\left({p}_{j}^{i}\right)$$

where ${p}_{j}^{i}$ is the probability of instruction $j$ appearing at position $i$ in the sampled genotypes. The value is estimated by dividing the total number of genotypes with the given instruction $j$ at the site by the total number of genotypes in the population.

$${{p}_{j}^{i}}= \frac{\text{Number of genotypes with instruction $j$ at genomic site $i$}}{\text{Total number of genotypes in the population}}$$

### Phenotypic heterogeneity of the population

The phenotype of an organism is the set of tasks it can (or cannot) perform when supplied with inputs. The nine different tasks available in our simulations can thus define a total of 512 (2^9^) phenotypes—the number of possible combinations of these nine binary choices. To calculate the phenotypic heterogeneity of a population, the organisms under each of these phenotypes were counted. The Shannon entropy of this distribution gives the phenotypic heterogeneity of the population. The minimum possible value of zero is attained when the entire population consists of a single phenotype. The maximum heterogeneity is achieved when the population is evenly distributed between all 512 phenotypes.

### Software

Version 2.14.0 of Avida was used for these simulations. Automation of the analysis, data retrieval and cleanup were done using Python version 3.8.2 (with numpy 1.19.0 and matplotlib 3.2.2). Data analysis, statistics and plotting was performed using R version 4.0.2 and the ggplot2 library (version 3.3.2). The details of the statistical tests are provided in Additional file 2. All these experiments were conducted on x86-64 machines running GNU/Linux kernel 4.15.0.

## Results

### Mutation rate regulates diversity in fitness acquisition and associated genotypes

We began by comparing the genotypes obtained after 100,000 updates of evolving the ancestral Avida organisms and plotted the distribution of fitness for genotypes obtained at high and low mutation rates (and at three resource availability values and three population sizes) in Fig. 2. For the low mutation rate, five major peaks were observed in the fitness distribution (Fig. 2a, peaks denoted as “L0”, “L1”, “L2”, “L3” and “L4”—labelled in ascending order of their median fitness). The peaks were found to be higher in fitness than that of the ancestral organism. For the high mutation rates, we observed four major peaks (Fig. 2b, denoted “H0”, “H1”, “H2”, and “H3”—labelled in ascending order of their median fitness). Peak H0 was lower and peaks H1–4 were higher in fitness compared to the ancestral type. We note that only a single dominant peak was observed for most runs (Additional file 1: Fig. S1). Therefore, each of the fitness peaks likely represented a stochastically chosen stable state and a population evolved into one of these states with different probabilities as the conditions were varied. To verify this, we plotted the distribution of fitness at intermediate times and verified that the number of generations used here gave a stable, non-shifting distribution by the end of the run (Additional file 1: Fig. S2). Larger world sizes (carrying capacities) gave a non-unimodal distribution of fitness indicating a mode of existence where multiple dominant genotypes can coexist. We tested for unimodality of the fitness distribution by calculating the Hartigan dip-test value with the alternative hypothesis that the distribution is multimodal [27]. Carrying capacities over 500 gave populations that were significantly multimodal (Additional file 1: Fig. S3). In summary, the mutation rate determined the set of major genotypic fitness values that emerge at the end of evolutionary runs. The resource availability and population size only affected the relative probability of genotypes attaining one of these values (Additional file 1: Fig. S4). The representative genotypes were isolated from a small region around the peak maxima as given in Additional file 1: Table S2.

### Genomes that evolved at a low mutation rate exhibit differences in genome replication rate and metabolism

**Fig. 3:**
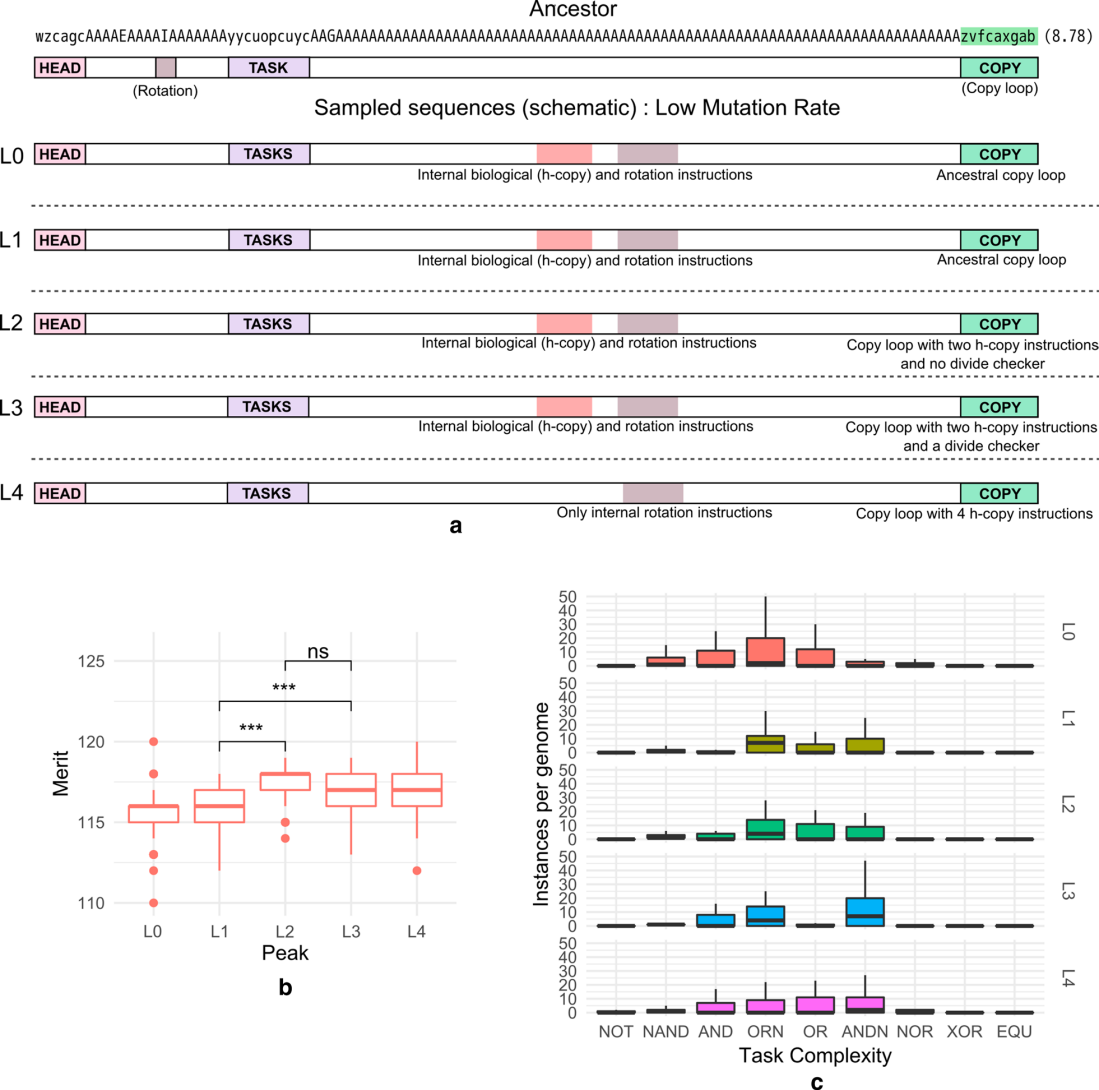
**a** Schematic depiction of key genomic elements from the ancestral organism (top) and representative organisms from L0–L4 peaks obtained from simulations at the low mutation rate (see Fig. 1a). The copy-loop (highlighted in green) is the major source of difference between these sequences. Some of the sample sequences from each of these peaks are given in Additional file 1: Fig. S13. The fitness ranges used to isolate these peaks are given in Additional file 1: Table S2. **b** Box plots depicting merits (speed of the organism’s virtual CPU, a proxy for metabolic activity of the genotype) of 100 genotypes sampled from each of L0–L4 on the fitness distribution of simulations performed at a low mutation rate. Genomes from L0 and L1 are metabolically less active compared to L2, L3, and L4 (statistical significance measured using the two-tailed unpaired Wilcoxon test; ***p < 0.001). **c** Task complexities for genomes (estimated roughly by the number of “NAND” instructions required to perform a task, increases from left to right) from L0–L4 peaks on the fitness distribution obtained from simulations at a low mutation rate. Higher fitness peaks are found to perform more complex metabolic tasks. The Y axis represents the number of times that a genotype belonging to these peaks performs a task over a single execution of the genome. The data for this figure is also given in Additional file 1: Table S3

We examined the genotypes cognate to the peaks in the fitness distribution to identify the differences in sequence that accompanied their evolution. The predominant difference between the genomes from the corresponding peaks (evolved at low mutation rate) was found in their genome replicative machinery (Fig. 3a): the h-copy (v) instructions (which may be comparable to the prereplication complexes that regulate the speed of the replication fork movement [28]) were present in different numbers in the copy-loops of genomes across the peaks. These instructions are responsible for copying the genome instruction-by-instruction and a larger number in the copy-loop denotes increased replication rate per cycle.

Organisms from peaks L0 and L1 showed a copy-loop identical to the ancestor, containing a single h-copy (v) instruction and an if-label instruction (f) that validated the completion of the genome duplication process and only then allowed the offspring genome to segregate from the parent. Organisms from L2 peak used two h-copy instructions which allowed them to copy two genomic instructions every iteration, but did not have a validation system, potentially allowing the birth of partially copied non-viable genotypes. The overall effect was an increase in fitness, but organisms with these genotypes nevertheless had a lower fitness relative to the ones that retain the validating if-label instruction as found in L3 peak. L4 peak genomes (which showed the highest fitness) were able to evade this requirement by incorporating a large number of h-copy instructions (generally four), essentially allowing them to rapidly copy the entire genome without offspring sequence validation. The low mutation case thus contrasts the differences in genome-copying robustness that evolves under different conditions.

In addition to differential replicative rates, we observed the duplication of elements present within the copy-loop and their transition to the non-copy-loop regions of the genotypes for all peaks except genomes of peak L4. However, measuring the marginal utility of these *internalized* elements showed that they did not contribute significantly to the fitness of the genotypes (Additional file 1: Fig. S5; median values for all L-peaks were found to be lesser than 0.05, indicating that the addition of these internalizations added less than 5% fitness advantage to the sequences).

**Fig. 4:**
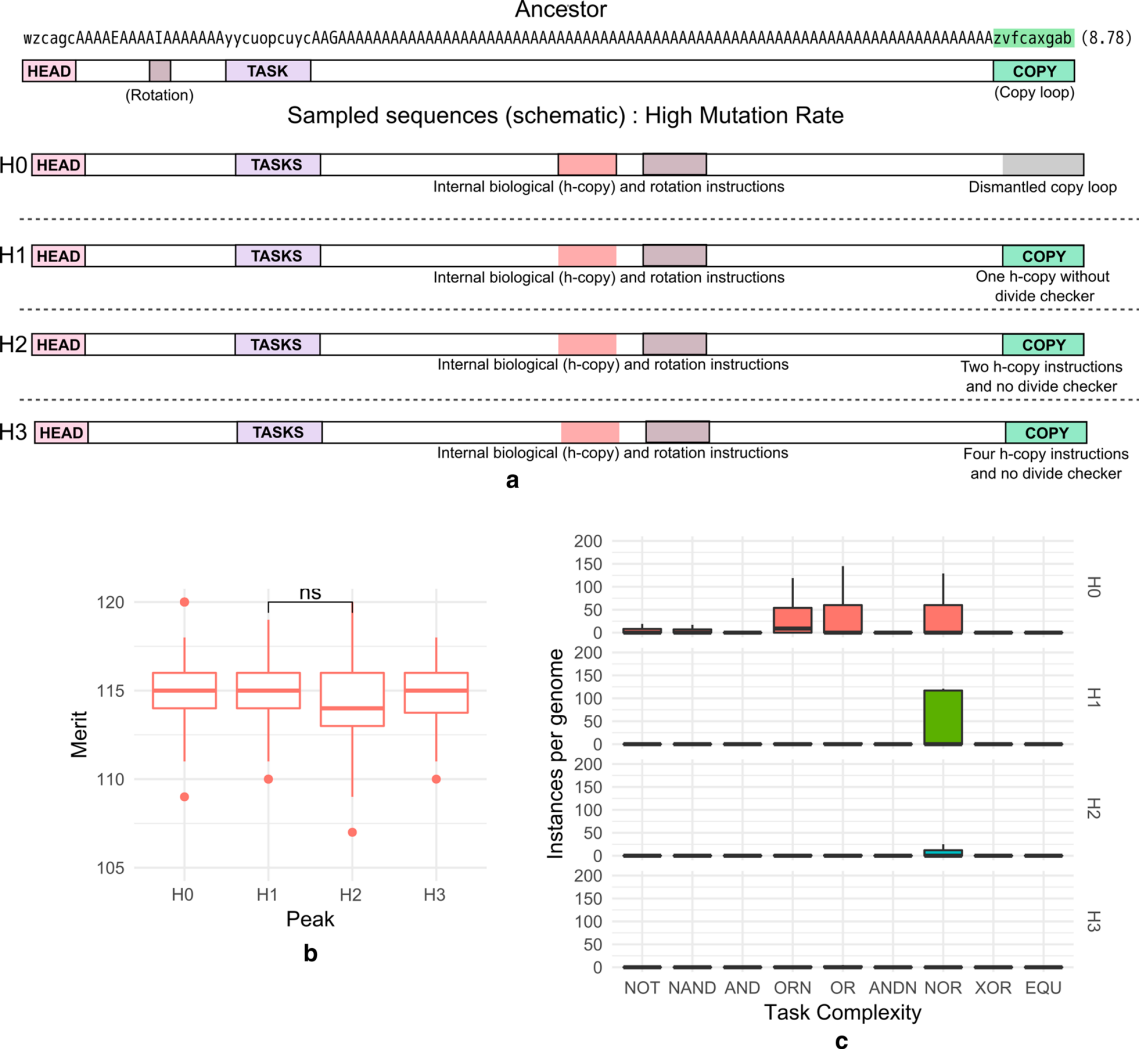
**a** Schematic depiction of key genomic elements from the ancestral organism (top) and representative organisms from H0–H3 peaks obtained from simulations at the high mutation rate (see Fig. 1b). The copy-loop is the first point of difference between these sequences. H0 genotypes have a copy-loop devoid of the ancestral copying functionality. Some of the sample sequences from each of these peaks are given in Additional file 1: Fig. S14. The fitness ranges used to isolate these peaks are given in Additional file 1: Table S2. **b** Box plots depicting merits (speed of the organism’s virtual CPU, a proxy for metabolic activity of the genotype) of 100 genotypes sampled from each of H0–H3 on the fitness distribution of simulations performed at a high mutation rate (statistical significance measured using the non-parametric Wilcoxon test). **c** Task complexities for genomes (estimated roughly by the number of “NAND” instructions required to perform a task, increases from left to right) from H0–H3 on the fitness distribution obtained from simulations at high mutation rate. The Y axis represents the number of times that a genotype belonging to these peaks performs a task over a single execution of the genome. Note that peak H3 does not perform any of the metabolic tasks but instead relies on a vastly improved replicative machinery (four h-copy per cycle) to acquire a very high fitness. The data for this figure is also given in Additional file 1: Table S3

The organisms from different peaks were also observed to exhibit differences in their capacity for utilization of resources (metabolism). L0 and L1 organisms acquired a lower cumulative median merit than those from peaks L2, L3, and L4 (Fig. 3b). The complexity of tasks (We define complexity of a task as the minimum number of NAND instructions required in the genome to execute the task) performed showed a progressive increase across organisms from peaks L0 to L4 (Fig. 3c). Figures 3c and 4c plot the tasks in the order of increasing complexity along the x-axis.

These results led us to investigate whether changes in the copy-loop contributed to determination of fitness and merit for the evolved genotypes. Replacement of the copy-loop in a representative genome from peak L0 with a copy-loop containing multiple h-copy instructions (from a L4 genotype) instantaneously increased the fitness to an intermediate value (Additional file 1: Fig. S7). Allowing the population to evolve, increased the fitness to L4 fitness levels. The merit on the other hand, increased instantaneously to the L4 value, confirming that the nature of the copy-loop determines the metabolic capacity of the organisms. A reverse experiment (transplant of L0 copy-loop into L4 genomes) also showed a similar reliance of fitness and merit on the replicative region of the genome (Additional file 1: Fig. S8). Specific genomic ‘chimerization’ influenced the viability of the organisms: although all L4 organisms with L0 copy-loops were viable, addition of L4 copy-loop to L0 organisms rapidly brought down the viability by 80% (viability is defined as the percentage of a set of genotypes that are able to replicate under high resource conditions when release in an Avida world).

### Genomes that evolved at a high mutation rate exhibit differences in evolutionary innovations

In keeping with the analysis above, genotypes associated with the four fitness peaks observed for evolution under a high mutation rate were analyzed for inter-sequence differences (Fig. 4a). Peak H0 genotypes showed a loss of the ancestral copy-loop at the end of the genome and a relinquishment of copying activities to instructions in the interior of the genome (as was also observed in the fitness peaks of the low mutation rate runs). The change in fitness that two sample genotypes from this peak experienced over evolutionary time showed that the decline was concomitant with the occurrence of a mutation that removed the h-search instruction (Additional file 1: Fig. S9a). In spite of the breakdown of the vital ancestral copy-loop, these organisms survived due to the presence of replicative elements in the interior of their genome (removal of such copy-loop elements led to loss of fitness for these genotypes; Additional file 1: Fig. S9b). Interestingly, the appearance of internal copying instructions was found to temporally precede the loss of ancestral copy-loop functionality and is evidenced in the genomes of organisms from all fitness peaks except for the L4. Such instruction duplications may therefore be part of chromosomal changes that do not by themselves alter fitness but prevent extinctions due to copy loop-disruptions [29].

H1 genotypes retained a copy-loop consisting of only the basic replication instructions (h-copy and h-divide) required for copying but did not implement an if-label instruction that allows detection of complete copying. Genotypes from H2 and H3 showed progressively greater h-copy instruction numbers in their copy-loop suggesting their higher fitness was a result of an enhanced rate of replication. Metabolically, peak H0, H1, H2 and H3 genomes were found to be almost equivalent in merit to each other (Fig. 4b, note that the measurement of merit still takes place in a test CPU and thus cannot account for information sharing as is seen in H1 genomes). H0 and H1 performed a very large number of metabolic tasks per cycle (compared to the genomes obtained at a low mutation rate). In H2 and H3, a large number of metabolic task executions were absent, but high replication rates were generated owing to the inclusion of h-copy instructions (Fig. 4c).

**Fig. 5:**
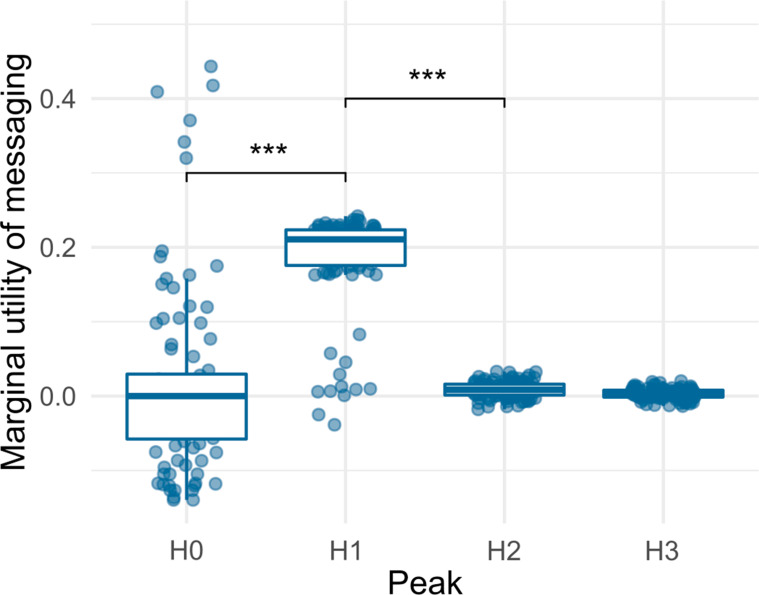
Box plot showing marginal utility of signaling for genomes (fractional addition to fitness provided by messaging, see "Methods") sampled from peaks H0–H3 of the fitness distribution obtained from simulations at a high mutation rate (see Fig. 1b). Only peak H1 utilizes messaging as a facilitator for acquiring a higher fitness (significance measured using the two-tailed non-parametric Wilcoxon test ***p < 0.001)

We observed that in addition to changes in copy-loop sequences and metabolic task elements, signaling (information sharing) instructions were also found in genotypes representative of peak H1, H2 and H3. On sampling and testing the utility of signaling in these genotypes (see "Methods"), we found that messaging contributed significantly to the fitness acquired by genotypes associated with peak H1, but not H2 and H3 (Fig. 5). Interestingly, the increase in fitness over the ancestral type for these (H1) genomes was approximately equal to the contribution from signaling (~ 20%).

**Fig. 6:**
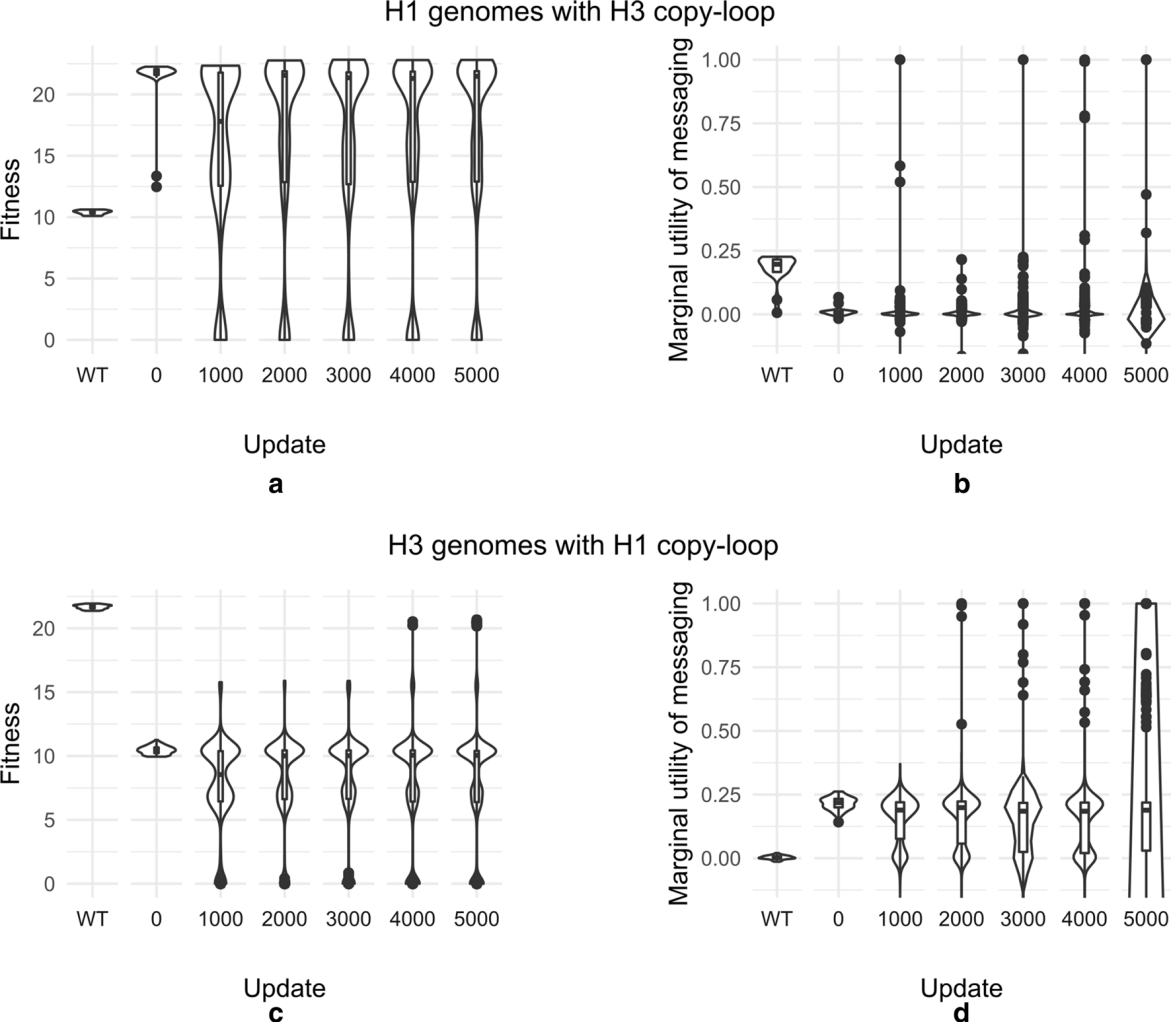
Violin plots showing **a** fitness and** b** marginal utility of messaging of genomes sampled from H1 upon their endogenous copy loop being replaced with H3 copy-loop and evolution for 5000 updates.** c** Fitness and** d** marginal utility of messaging of genomes sampled from H3 upon their endogenous copy loop being replaced with H1 copy-loop and evolution for 5000 updates. Updates on the x-axis represent the evolutionary time for which the hybrid genotypes were evolved. “WT” denotes these measures for the recipient genomes without copy-loop replacement

When the copy-loop of H0 genotypes was replaced with that of a peak H3 genotype, and the hybrid genotype was allowed to evolve, we did not observe an instantaneous increase in fitness: rather, the initial unchanged median fitness gave rise to multiple genotypes both lower and higher in fitness than the initial hybrid (Additional file 1: Fig. S10). H1 genotypes with their copy-loops replaced by H3 copy-loops showed an instant increase in fitness which diversified over evolutionary time (Fig. 6a). The viability, just as in the low mutation rate case, was affected as a result of the change and only 20% organisms survived. Removal of the cognate copy-loop from H1 and replacement with H3 copy-loop dropped the marginal utility of messaging to 0 (Fig. 6b). Consistent again with the low mutation rates, 100% of peak H3 genotypes, upon their copy-loops being replaced by the H1 counterpart, evolved to fitness levels congruent with H1 genotypes (Fig. 6c). To our surprise, an increased marginal utility of messaging was found to be conferred by the addition of the H1 copy-loop to genotypes from H3 (Fig. 6d). These transplant experiments indicated that the copy-loop played a major role in determining the benefits that an organism accrues through signaling.

### Populations dominated by signaling genotypes are more genetically and phenotypically heterogeneous

**Fig. 7:**
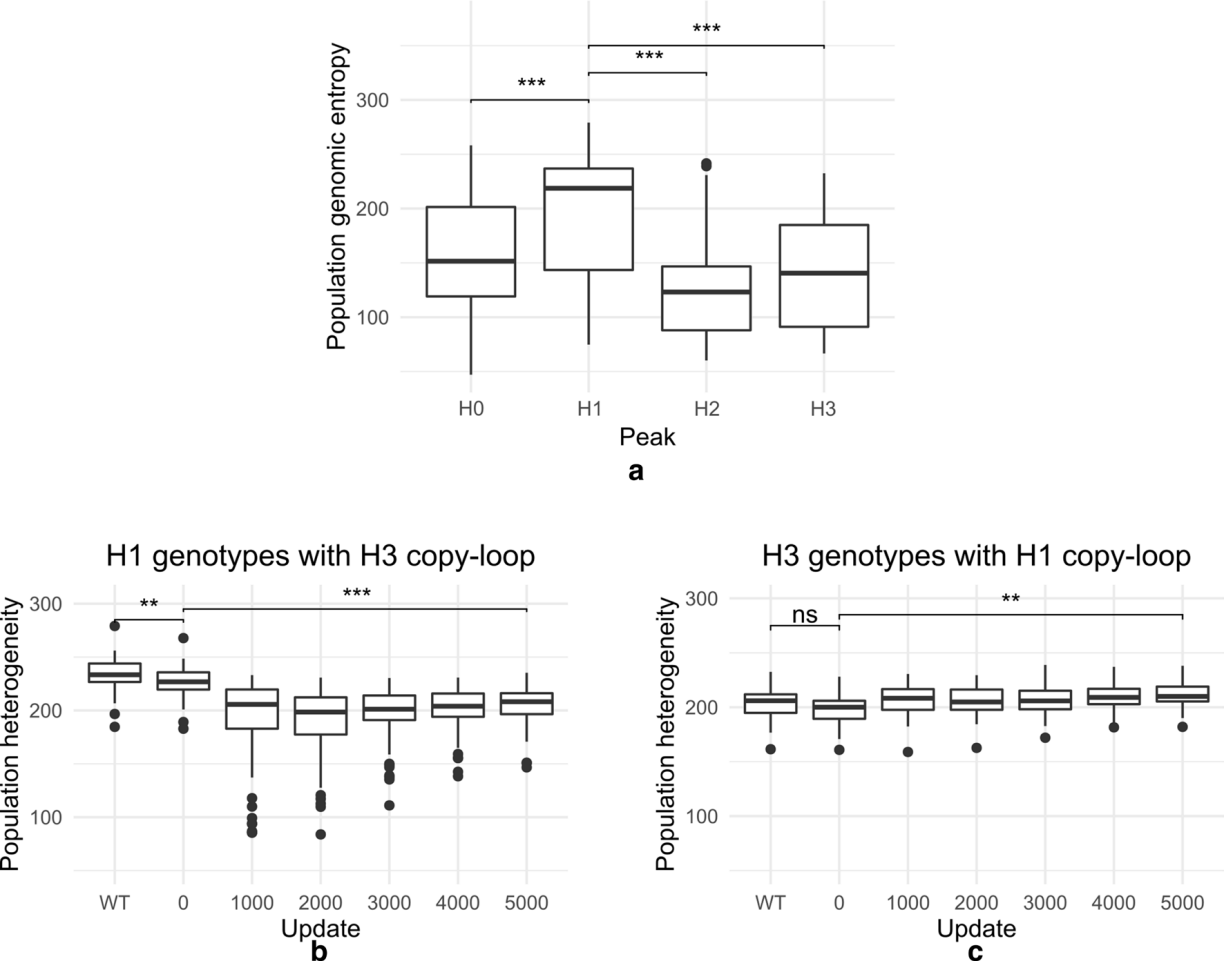
**a** Box plots showing genomic heterogeneity within populations belonging to H0–H3 peaks of the fitness distribution obtained from simulations at a high mutation rate (see Fig. 2b). **b** Change in population heterogeneity when populations dominated by H1 are transplanted with H3 copy-loop and evolved for 5000 updates (Populations evolved under size 500 and 1000 k resource availability). **c** Change in population heterogeneity when populations dominated by H3 are transplanted with H1 copy-loop and evolved for 5000 updates. Population heterogeneity is measured by calculating the sum of per-site genomic entropies (see “Methods”, significance calculated using the non-parametric Wilcoxon test; **p < 0.01, ***p < 0.001). “WT” denotes heterogeneity of the recipient population without copy-loop replacement

We asked whether populations dominated by the signaling positive peak H1 genotypes had greater genetic heterogeneity. Shannon entropic measurements made across sequence alignments of populations that evolved under high mutation rates showed that signaling populations were genetically more heterogeneous compared with populations from the other peaks associated with a high mutation rate (Fig. 7). This variation was seen to be almost invariant across resource levels and population sizes (Additional file 1: Fig. S11). To check whether this difference in genotypic heterogeneity was specific to the mechanism of fitness acquisition, we transplanted copy-loops from H1 and H3 into each other’s genotypes. To ensure that the process of transplant itself does not introduce changes in heterogeneity, we performed a control run transplanting H1 populations with a H1 copy-loop (Additional file 1: Fig. S12). Addition of H3 copy-loop to H1 populations decreased the population heterogeneity to H3 levels over 5000 updates of evolution (Fig. 7b). A reverse experiment of replacing H3 populations with H1 copy-loop significantly increased the population heterogeneities as well (Fig. 7c).

**Fig. 8:**
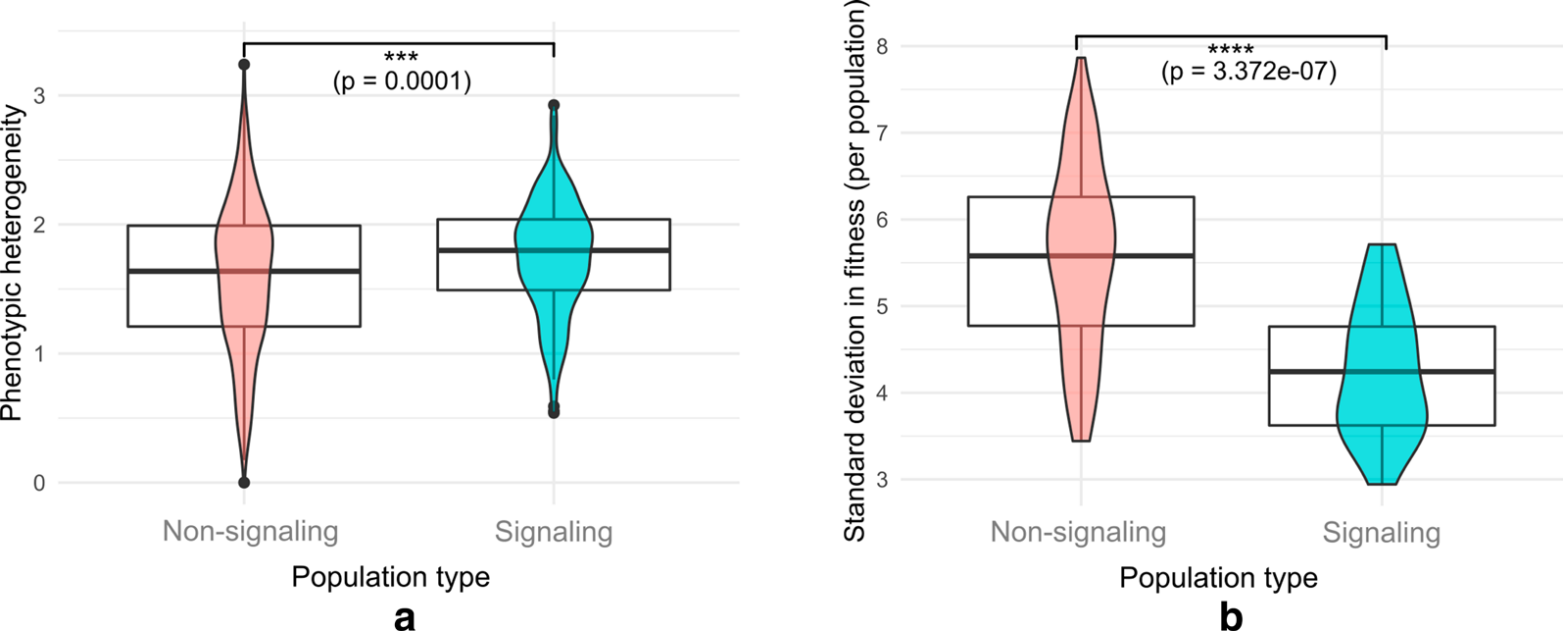
**a** Phenotypic heterogeneities for non-signaling and signaling populations. (significance calculated using the Welch’s t-test; ***p < 0.001). **b** Standard deviation in fitness for non-signaling and signaling populations

Isolated signaling positive populations were also found to be phenotypically more heterogeneous than their non-signaling counterparts under all conditions (Fig. 8a). Interestingly, the spread in fitness was found to be smaller for signaling populations, indicating that the phenotypic heterogeneity did not manifest as a heterogeneity in fitness outcomes (Fig. 8).

### Population size and resource levels affect the frequency of occurrence of signaling populations

**Fig. 9:**
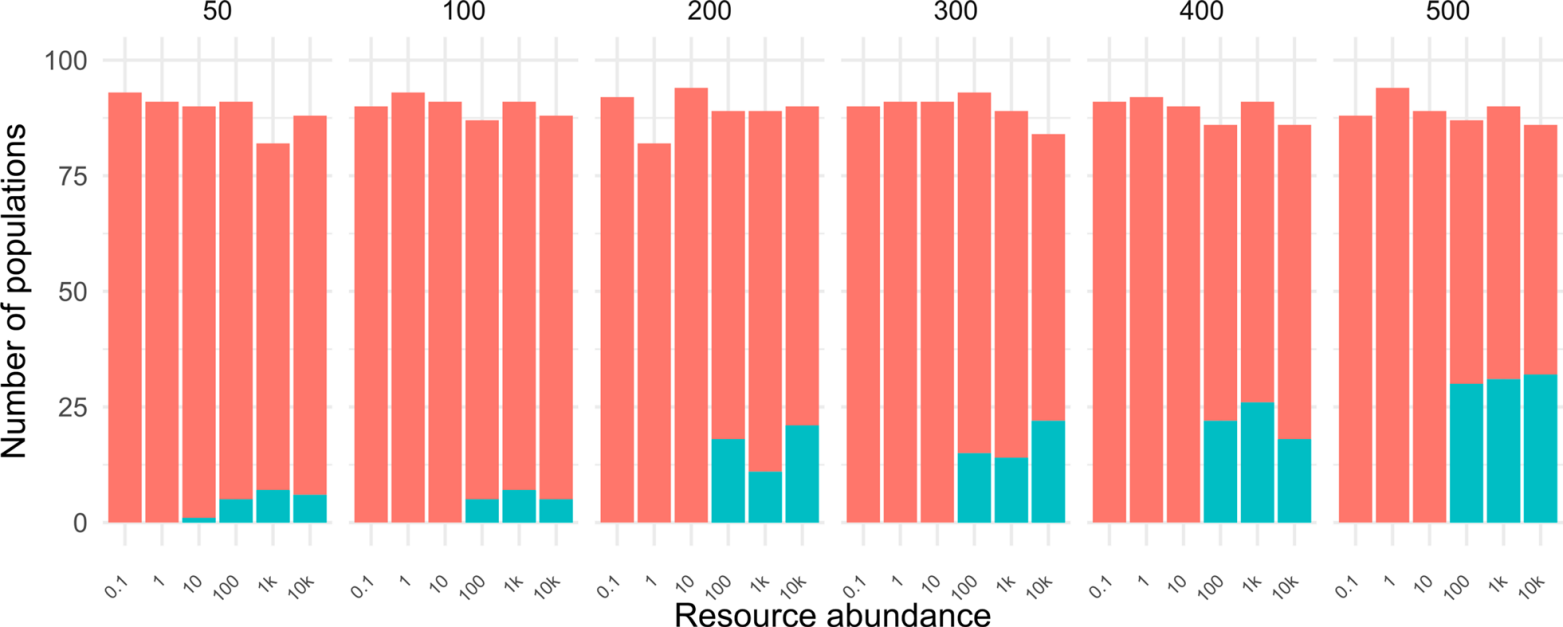
Variation in incidence of signaling populations as resource abundance and population size is varied. Signaling positive populations (blue) are involved in metabolic signaling and their frequency is seen to increase as the population sizes are increased. An increase in resource abundance also increases the effect of population size on their incidence. Lower populations give way to signaling at lower resource abundances. A total of 100 replicate populations were used for these experiments, the deficit below 100 indicates the number of extinctions before the populations could evolve to the end of the run

We plotted the incidence of signaling positive populations for different population sizes and resource abundances. We observed a steady increase in the probability of occurrence of the signaling populations as the population size was increased from 50 to 500 (Fig. 9). Resource abundance also showed a positive effect on the emergence of the signaling population for all population sizes (Fig. 9).

## Discussion

In this work, we have explored the interplay between the environment and the driving force of phenotypic variation: the mutation rate, in the exploration of an adaptive landscape by a population of digital organisms. It is increasingly being recognized that the effect of mutation rate on fitness needs to be examined in the context of the environment in which evolution takes place [30, 31]. In this manuscript, we consider two environmental parameters: the carrying capacity and resource levels. A positive effect of population size on fitness can be explained by the higher probability of mutants appearing in the population; in fact under high mutation rate, bigger population size allows for greater exploration of the fitness landscape and fixation of genotypes that may otherwise never appear by mutational variation in a smaller population [32]. At the same time, larger populations are less susceptible to drift and can thus allow selective effects to dominate. We see that population size and resource abundance influence fitness in complex and distinct ways under high and low mutation rates. Higher values of both population size and resource abundance do favor greater frequencies of reproductively efficient high fitness genotypes (specifically L3 but also L2) over relatively inefficient counterparts (L1). When the mutation rate is high, the frequency of suboptimal fitness genotype (H1), acquired through signaling under high mutation rates, also shows a direct correlation with population size and resource levels. These observations establish the importance of the interdependence between the three input variables we have chosen in the study and show how fitness levels, as well as the mode of fitness acquisition are nuanced multidimensional outputs of the genotype and the environment within which it evolves.

High mutation rates allow the acquisition of fitness through signaling between organisms (peak H1). Even in this case, messaging instructions that allow for signaling appear in other genotypes but their contribution to fitness for such genotypes is minimal, compared to H1 genotype whose fitness depends on such instructions. These observations are consistent with the robust-yet-fragile hypothesis extended for complex adaptive systems, wherein fitness of genotypes could evolve robustness against a contingency while remaining sensitive to others [33]. It is pertinent to note that signaling is associated with the presence within populations of greater genetic and phenotypic heterogeneity than the other three peaks: the suboptimal nature of fitness levels for the H1 signaling positive state can be interpreted to be due to a weaker selection against fitness-impairing mutants when they arise in this population. Our results suggest how signaling may evolve within discrete transitory cancer populations, such as metastatic spheroids, wherein a tradeoff between fitness and replicative efficiency on the one hand, and the emergence of heterogeneity and signaling on the other, may likely influence therapeutic strategies [2, 18].

Whereas our evolutionary framework captures sequence evolution and its correlation with evolution of genotypes fairly well, we wish to emphasize a few notable limitations. An important one is the absence of a developmental time scale and phenotypic (morphological and behavioral) traits associated with it. Incorporation of this time scale would allow the testing of the effects of nonadaptive plasticity of the phenotype on its evolution within populations [34, 35] and in a broader context, the effect of development on the evolution of phenotype [36–39]. Even if the developmental time scale is not explicitly incorporated in our framework, our demonstration of a cellular-signaling dependent mode of evolution under high mutation rates and environment-permissive conditions provide insights into how multicellular organization could have emerged from unicellular life-histories [40, 41]. In fact, efforts to explain evolutionary bursts within phyla or rapid speciation predict higher rates of mutation or genomic evolution as causative to their occurrence [42–44].

The framework also implicitly assumes that the response to environmental changes is to be directly coded if at all within the genome—there is no secondary storage of information like epigenetic modifications or developmental encoding of information such as real cells in a structure. This disallows the potential to test environmental effects on epigenetic (used in the classical Waddington sense [45]) mechanisms, such as phenotypic plasticity that contribute to phenotypic evolution [46–48]. The evolutionary process in Avida can thus affect changes that take place at the scale of a single organismal lifetime to a greater extent than it does for real biological systems. We have also not allowed our simulations to run across huge population sizes: doing so may allow us to witness the dynamics of coexistent genotypic mutants, which we seldom see in our runs. However, our motivation in this study was to study evolution within smaller asexual populations, such as in the case of transitory metastatic niches of dividing cancer cells, and derive fundamental principles for their behaviors over long time scales [49]. It has not escaped our notice that high mutation rates engender the evolution of plastic, heterogeneous and cooperative populations, properties increasingly being attributed as fundamental to transitory malignant neoplasms [50, 51]. A more rigorous comparison between these two systems will be undertaken in the near future.

## Conclusions

We conclude by pointing out that our observations reinforce the fact that specific environmental variables play an important role in determining both the variety and the probability of evolutionary outcomes that arise within finite populations. In the background of a higher rate of mutation, such variables even facilitate the evolution of metabolic signaling within genetically heterogeneous organismal ensembles.

## Supplementary Information

**Additional file 1:** Supplementary information, figures, and tables.

**Additional file 2:** Details of statistics used in the paper.

## Data Availability

The datasets generated and/or analyzed during the current study are available in the GitHub repository, https://github.com/aVeryStrangeLoop/rblab_figures.

## Abbreviations

ATP: Adenosine triphosphate
NADPH: Nicotinamide adenine dinucleotide phosphate (reduced form)
KDE: Kernel density estimate
