# Supplementary material for: An interplay of resource availability, population size and mutation rate potentiates the evolution of metabolic signaling
Source: BMC Ecol Evol. 2021 Apr 7;21:52. doi: 10.1186/s12862-021-01782-0 (PMC8028831; doi:10.1186/s12862-021-01782-0)
Supplement: Supplementary file 1 — Additional file 1: Supplementary information, figures, and tables. [file 12862_2021_1782_MOESM1_ESM.docx]

## Additional information

*For “An interplay of resource availability, population size and mutation rate potentiates the evolution of metabolic signaling” by Bhaskar Kumawat and Ramray Bhat*

### S1. Organism life cycle and terminology

Each organism in Avida consists of the following components,

1. Three memory spaces, called **registers**, that can store a 5-bit number (the unit of information in Avida) each and are individually accessible by the organism – labelled AX, BX and CX
2. Two stacks that can store any number of 5-bit numbers in a first-in-last-out fashion, and
3. A **messaging buffer** which is a stack that can store messages received from neighbors. Numbers are inserted into registers when the organism executes a receive-msg instruction.
4. A CPU – composed of three heads and an instruction pointer - that executes the instructions that form the *genotype* of the organism.

The genotype is a linear series of instructions chosen from a given instruction set. In our experiments, the genotype length is restricted to 120 instruction-sites. In the case of uninterrupted execution, an **instruction pointer** moves along the genotype sequence and executes the instructions site-by-site. **Flow-control** instructions can change the location of the instruction pointer when suffixed with a **label** (composed to labelling instructions like nop-A, nop-B, or nop-C) to identify a site in the genome. This requires a second pointer, called the **flow-head**, to be at a specified location. The **read-head** and **write-head** are also pointers except they point to specific locations in the sequence and are static unless moved by a **mov-head** instruction. A **h-copy** instruction reads from the **read-head** and copies it to the **write-head** location. Once the entire genome has been copied once, it can be divided (**h-divide**) to form an offspring that is then placed in a site in the world being faced by the organism.

The Avida world is two-dimensional with each organism having eight neighbors. At a time, the organism points to one of these eight sites, called its **facing**. **Rotation** instructions can incrementally rotate the organism in either a clockwise or an anticlockwise direction. Offspring produced after a birth event are placed in the direction faced by the parent. Messaging through **send-msg** is also directional and the message is sent only to the nearest neighbor in the facing direction. The **bcast1** instruction can, however, send the message to all the eight nearest neighbors.

A detailed walkthrough of an example Avida organism can be found at https://github.com/devosoft/avida/wiki/Default-Ancestor-Guided-Tour

### S2. Complete list of instructions

The complete list of instructions used in our simulations is given here. The BX register is used by default for these instructions unless a nop modifier is specified after them. Register BX is a complement for AX, CX is a complement for BX, and register AX is a complement for CX.

| **Instruction(s)** | **Function** |
| --- | --- |
| nop-A, nop-B, nop-C | Modifier instructions, perform no function unless preceded by an instruction that requires labels or a modifier. |
| if-n-equ | Executes the next instruction only if specified registers have unequal numbers, otherwise skips it |
| if-less | Executes the next instruction only if specified register has a number less than its complement, otherwise skips it |
| if-label | Executes next instruction only if a specified template was just copied |
| mov-head | Moves either the read-head, write-head or the flow-head based on the succeeding nop modifier. |
| jmp-head | Moves instruction pointer by value specified in a register |
| get-head | Writes the position of the instruction pointer into a specified register |
| set-flow | Move the flow head to a position specified in a register. |
| shift-r | Shift all bits in a specified register to right |
| shift-l | Shift all bits in a specified register to left |
| inc | Increments specified register by one bit |
| dec | Decrements specified register by one bit |
| push | Copies value from register to top of current stack |
| pop | Removes value from top of current stack and puts it into a specified register |
| swap-stk | Toggles the currently active stack |
| swap | Swaps the value of a register with its complement |
| add | Adds the components of two registers |
| sub | Subtracts value of the complement of a specified register from its own |
| nand | Performs bitwise NAND on specified registers |
| h-copy | Copies an instruction from the read-head to the write-head |
| h-alloc | Allocates memory for offspring |
| h-divide | Divides off the offspring (between read and write head) and places it in a neighboring site in the world |
| IO | Outputs value of specified register and replaces it with a new input value from the environment |
| h-search | Finds a label and moves the flow-head after it |
| nop-X | Null instruction |
| send-msg | Sends the contents of a register and its complement to the faced neighbor’s message buffer |
| retrieve-msg | Retrieves message from message buffer to registers |
| bcast1 | Sends the contents of a register and its complement to all eight nearest neighbors |
| rotate-left-one | Rotates organism by one unit in anticlockwise direction |
| rotate-right-one | Rotates organism by one unit in clockwise direction |

### S3. Biological equivalence

1. Like any computational system, Avida has its own set of strengths and weaknesses in its ability to capture evolutionary processes. Unlike most analytical models or mean-field approaches, Avida explicitly models organism genomes – with a specialized set of instructions – allowing us to directly probe for the genetic determinants of processes and innovations that arise in populations. At the same time, the organisms in Avida are more susceptible to mutational effects because of the heightened role of each unit in the genome sequence, unlike DNA where a functional component is a gene rather than a single site. Avida is also designed to implement a simpler metabolic process model than what is seen in living systems. As such, there are certain areas where a one-to-one correspondence cannot be arrived at directly. To ease this process, we provide a list of processes in Avida that have clear correlates in living-systems.The genome is read and expressed in a non-simultaneous, sequential manner allowing for the possibility of complex regulatory processes to evolve. This also allows communicated information to be used for conditionally executing parts of the genome.
2. Replication occurs in a site-by-site manner and the resultant offspring is placed in proximity to the parent. Unlike genetic algorithms, replication is not mediated by the simulation but requires specific pathways to evolve in the organism which they then execute on Avida’s virtual substrate to reproduce. This is interestingly seen as the changing nature of copy-loop – the main replicating center – as conditions are varied.
3. The Avida metabolic model requires organisms performing computational work on input numbers and increased replication rates are awarded only if the computational work is done correctly – i.e. matches one of the task definitions. In living systems, energy to copy and reproduce is obtained by metabolizing chemical compounds which must be processed in an effective manner.
4. The messaging model can be seen as an information sharing pathway that allows individual cells to signal to their immediate neighbors similar to cell-surface signaling in biological organisms. This information allows neighbors to ease the effort required to process higher metabolites. At the same time, they can return this favor in form of the information about more profitable sources of energy in the environment.

The architecture of Avida organisms is much closer to computer programs due to the computational nature of the system. However, the above set of arguments allow us to draw more general arguments about the outcomes of the evolutionary process in a population of self-replicating, resource metabolizing organisms capable of information sharing – which Avida faithfully mimics.

## Additional Figures

*For “An interplay of resource availability, population size and mutation rate potentiates the evolution of metabolic signaling” by Bhaskar Kumawat and Ramray Bhat*


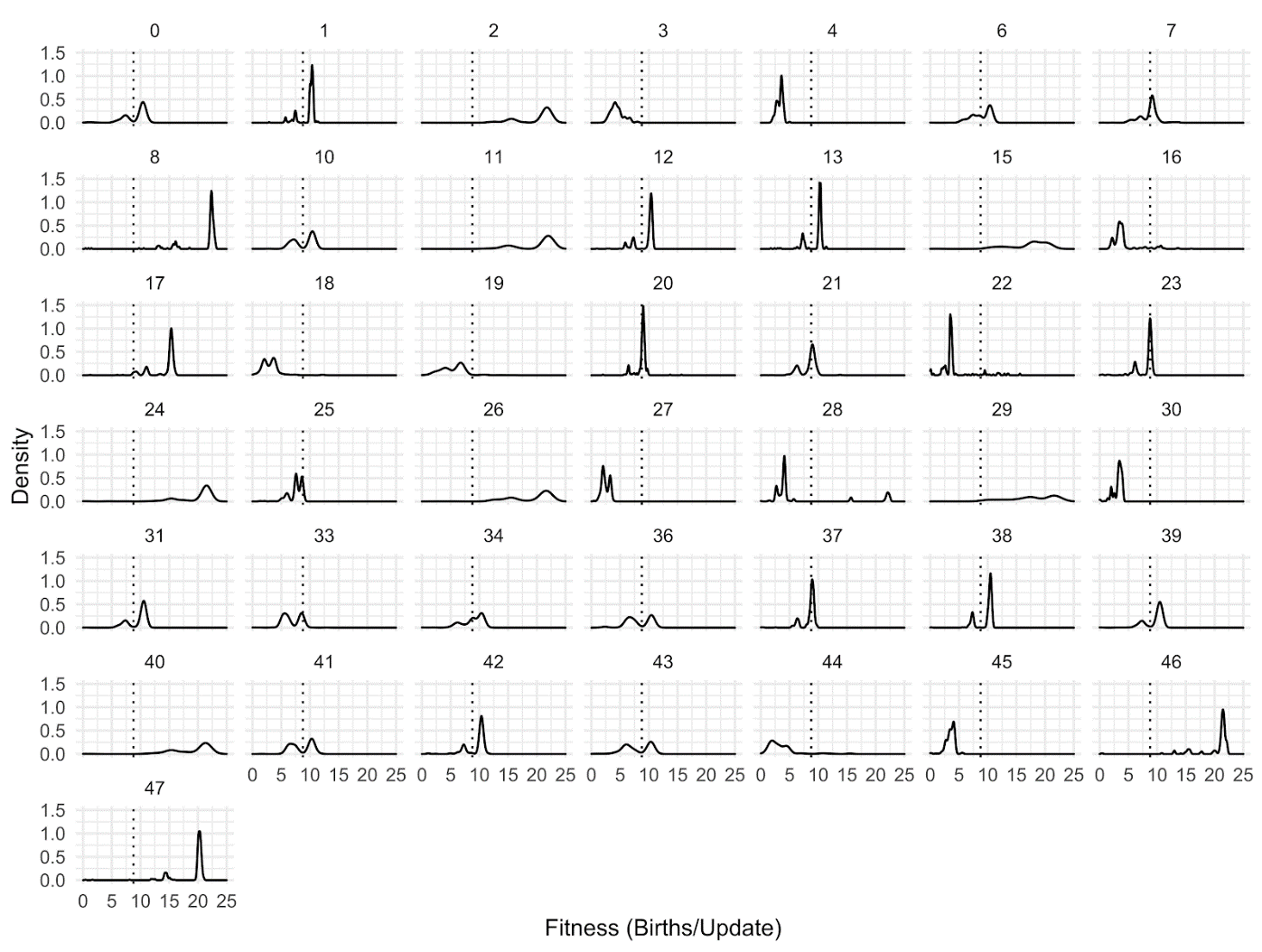


Figure S1. Fitness distributions for 48 populations obtained at a high mutation rate at a moderate resource availability (100k) and a high population size (500). Populations for two replicates went extinct early in the run and are not plotted here.


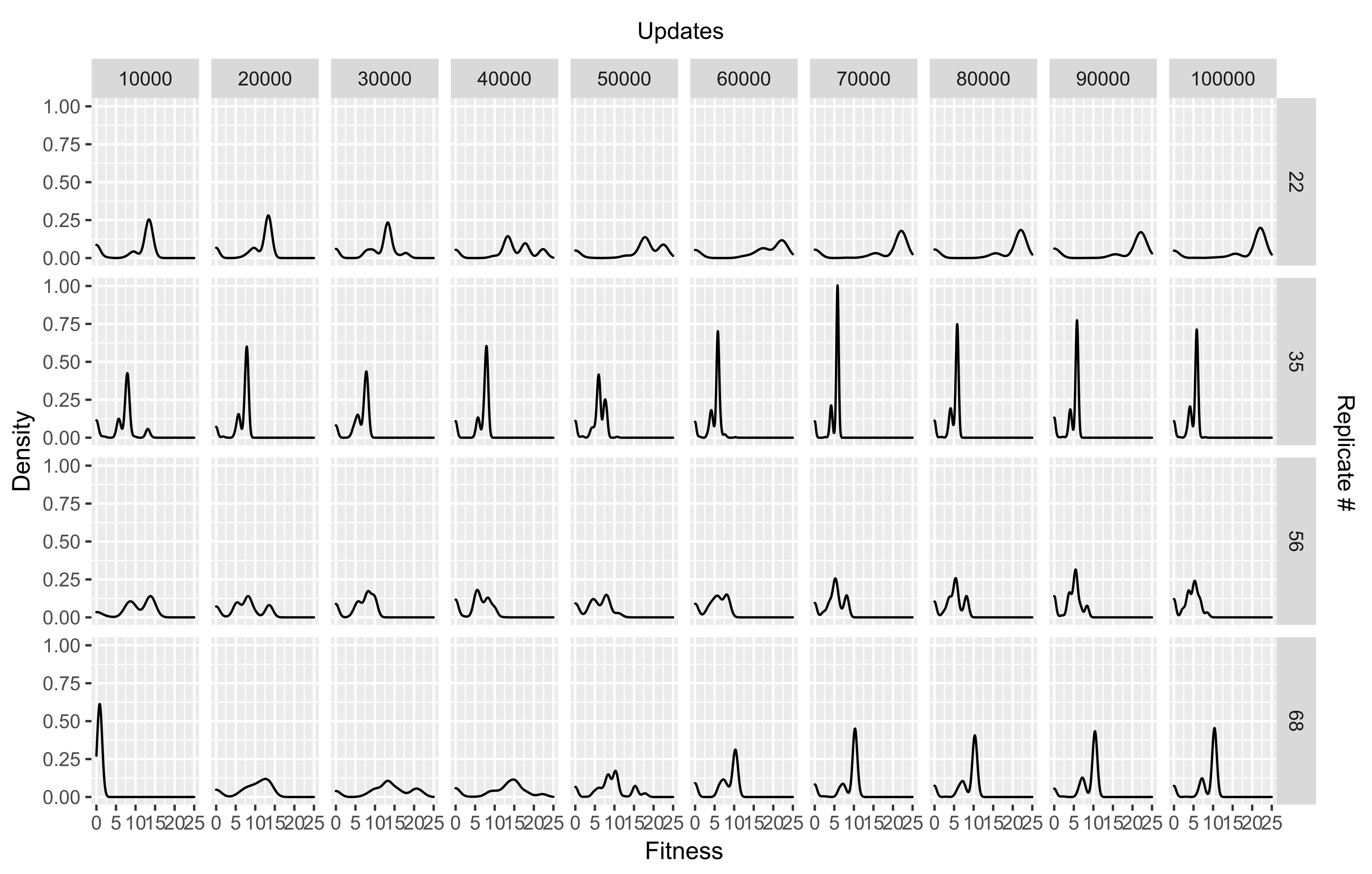


Figure S2. Change in fitness distribution over time for four illustrative replicates at population size 500 and resource abundance 1000k. As is seen here, the distributions stabilize by around 50,000 updates. The replicate populations evolve to distinct, unimodal, and stable fitness distributions.


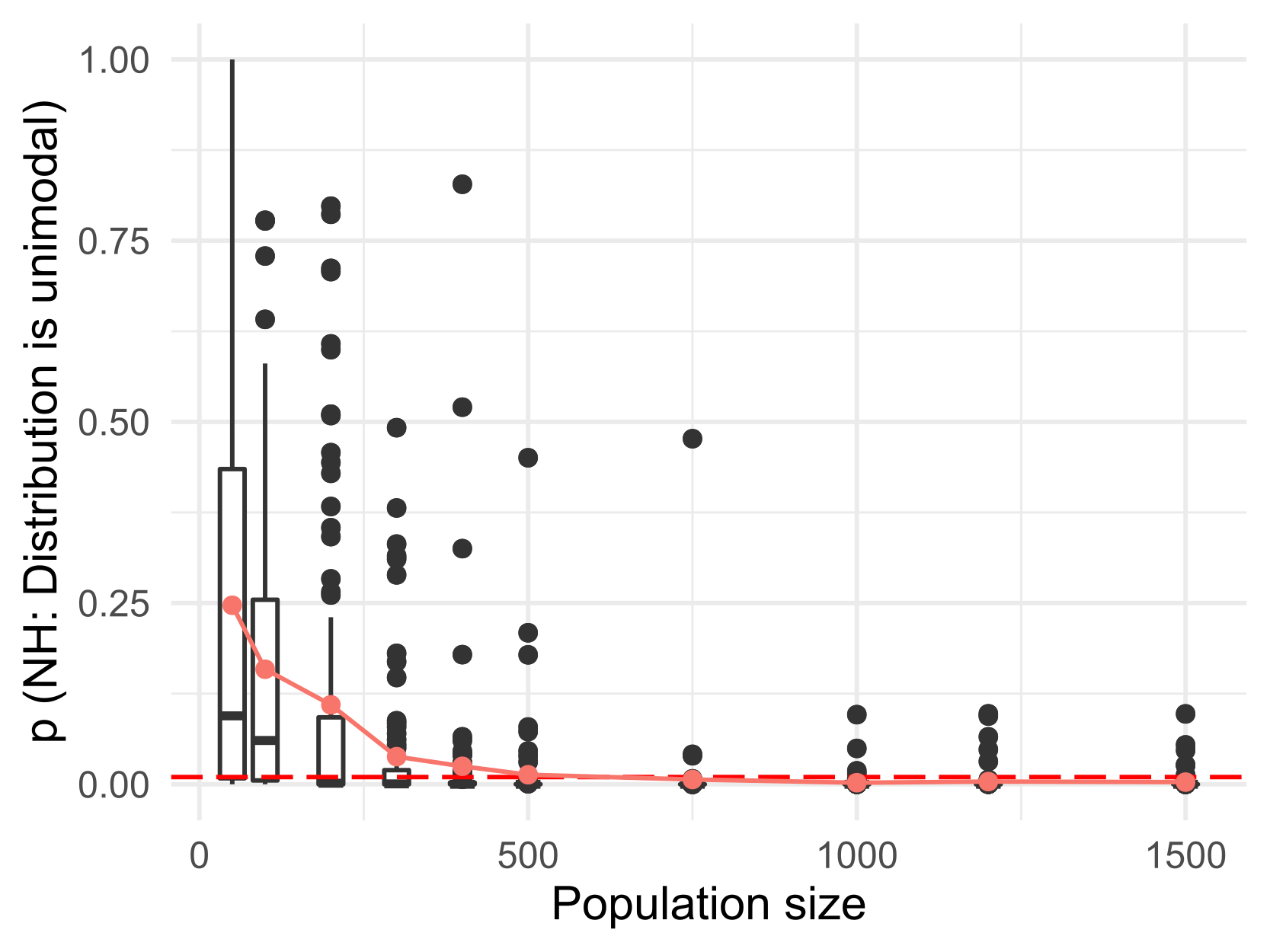


Figure S3. Hartigan dip-test for multimodality for fitness obtained at different population sizes (cumulative for all runs and resource abundances). The pink line denotes the mean of these values. Populations sizes above 500 are significantly non-unimodal (p<0.01).


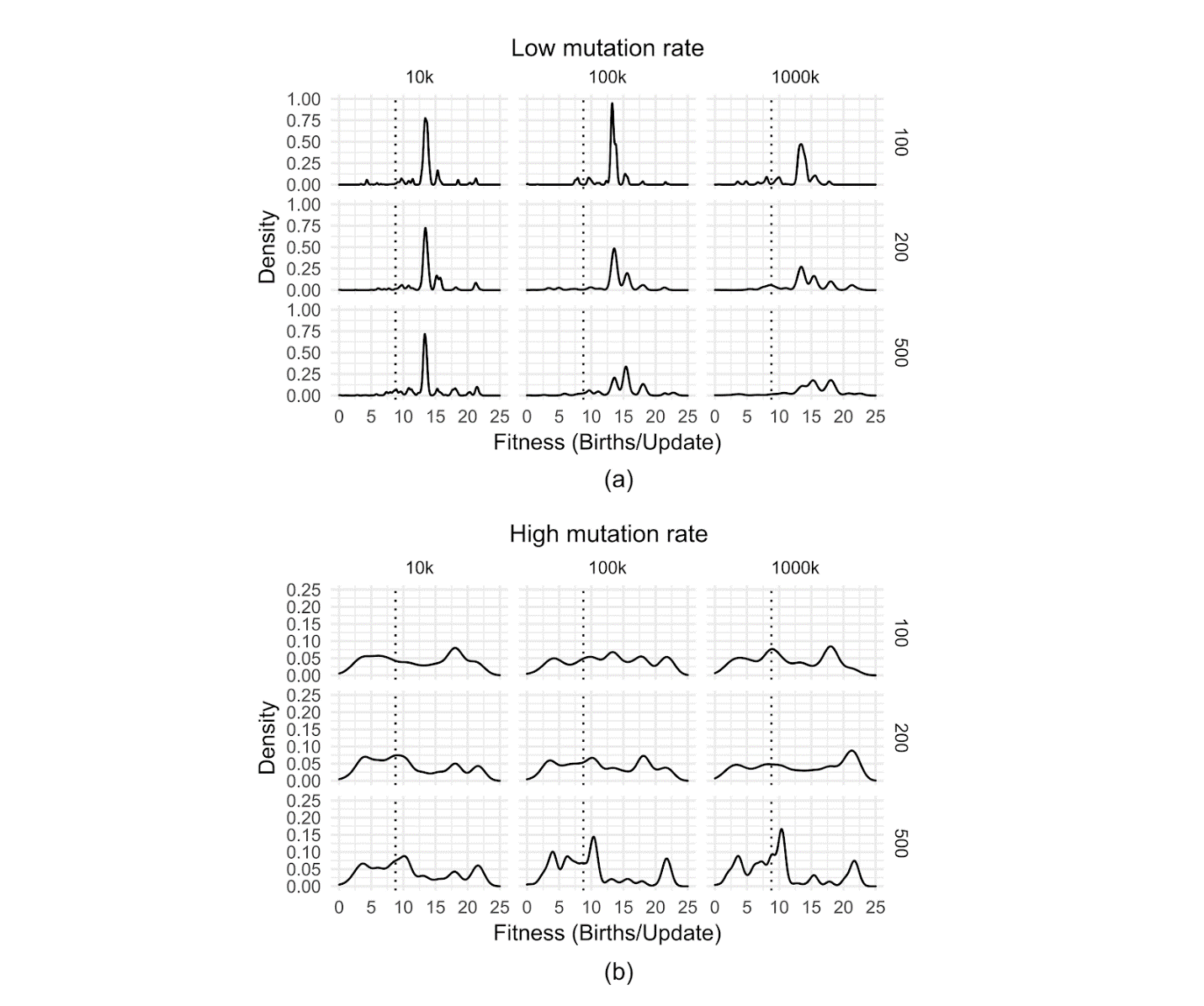


Figure S4. Fitness distributions of genotypes obtained for different values of resource availability and population size - (a) at a low mutation rate, and (b) at a high mutation rate.


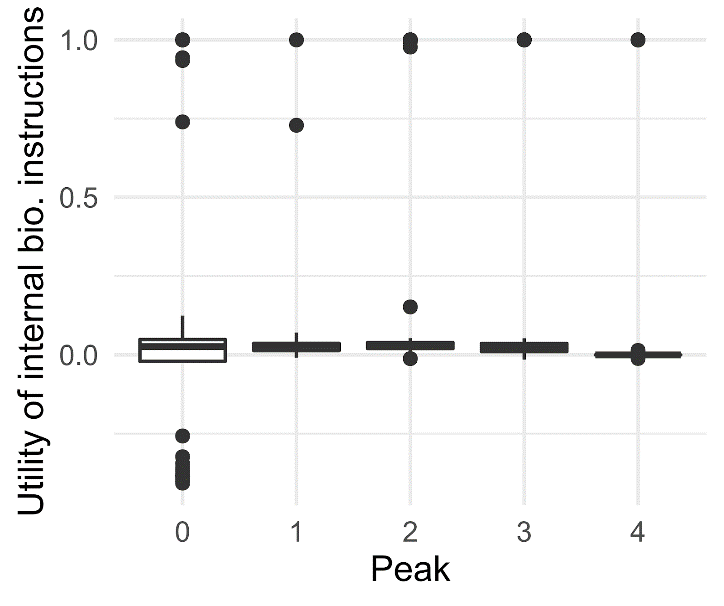


Figure S5. Marginal utility of internal biological instructions for genotypes belonging to different peaks at the low mutation rate (L0-L4).


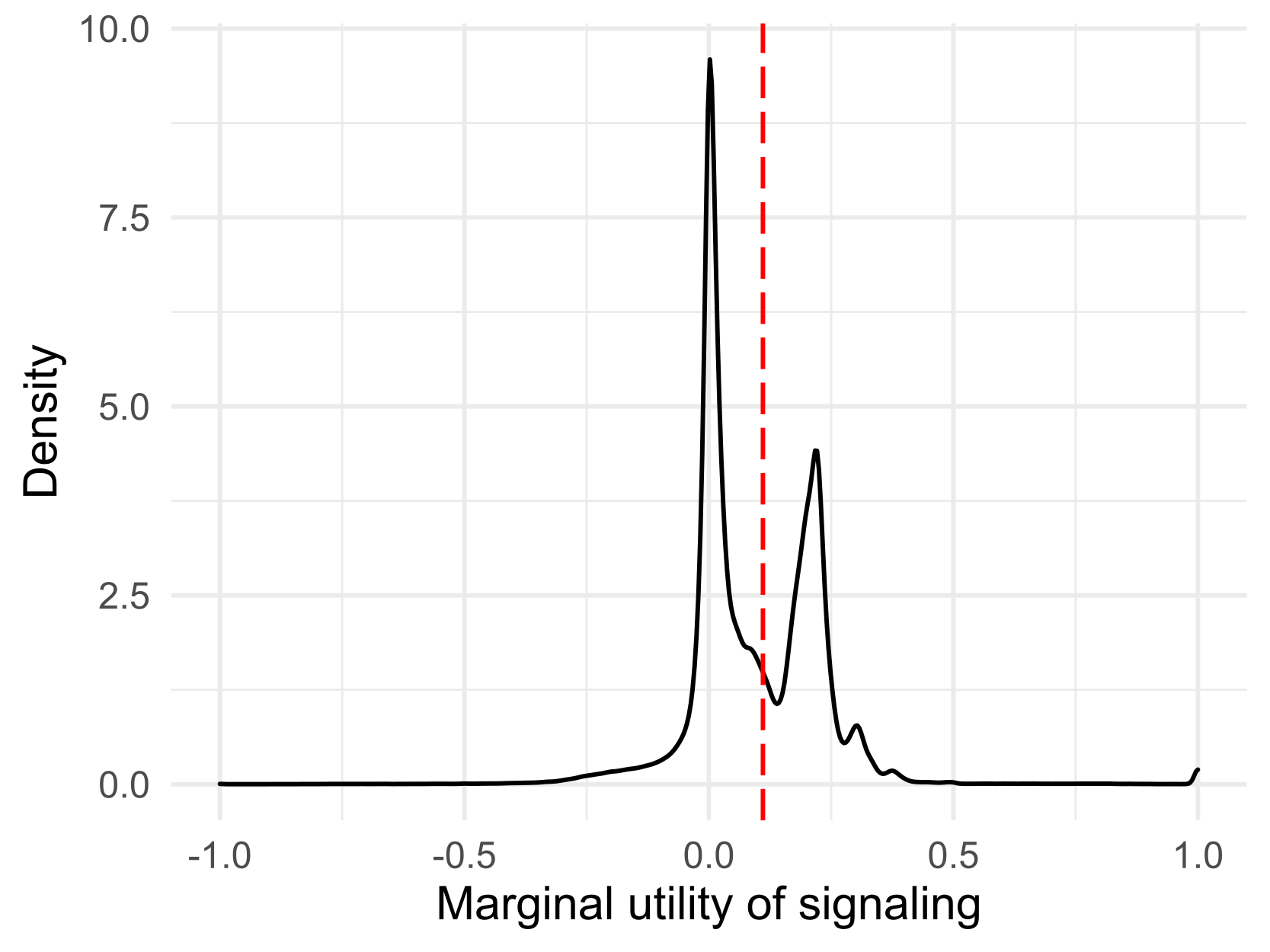


Figure S6. Combined distribution of marginal utility of signaling for all runs. This distribution shows two major peaks – one at zero consisting of non-signaling genotypes and one that consists of genotypes that obtain around 20% of their fitness through signaling. The midpoint of these peaks (red dotted line) is used as a threshold to distinguish signaling and non-signaling populations.


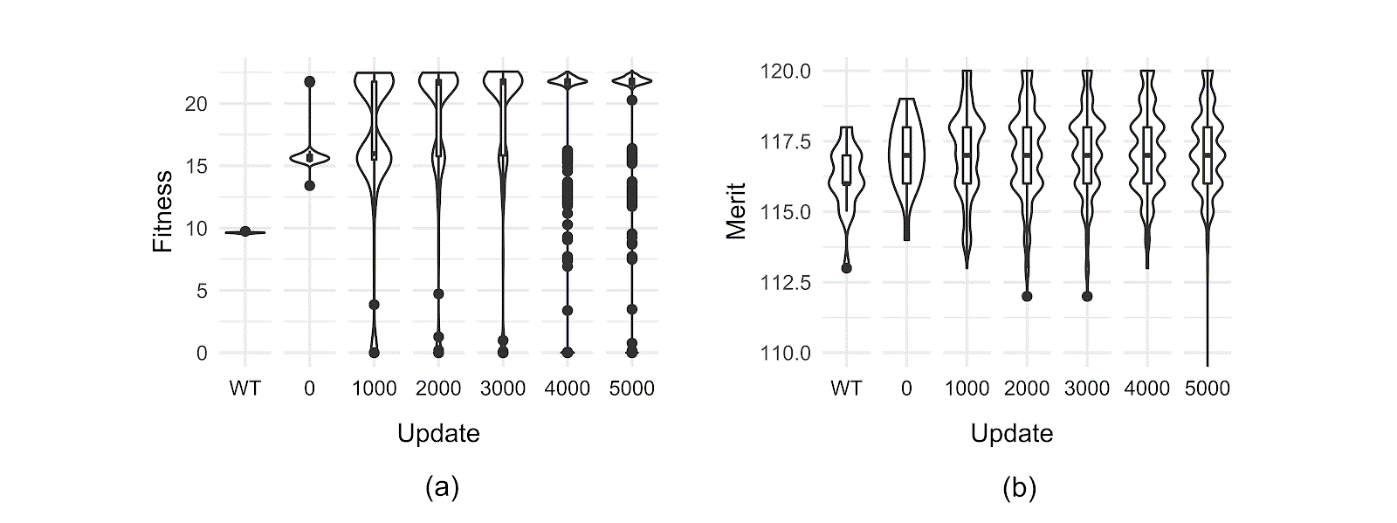


Figure S7. (a) Fitness and (b) merit evolution when recipient genomes sampled from peak L0 are transplanted with peak L4 copy-loop and evolved for different numbers of updates. Only viable recipient genomes are chosen (Viability ~ 19%). Updates on the x-axis is the evolutionary time for which the hybrid genotypes were evolved. “WT” denotes these measures for the recipient genomes without copy-loop replacement.


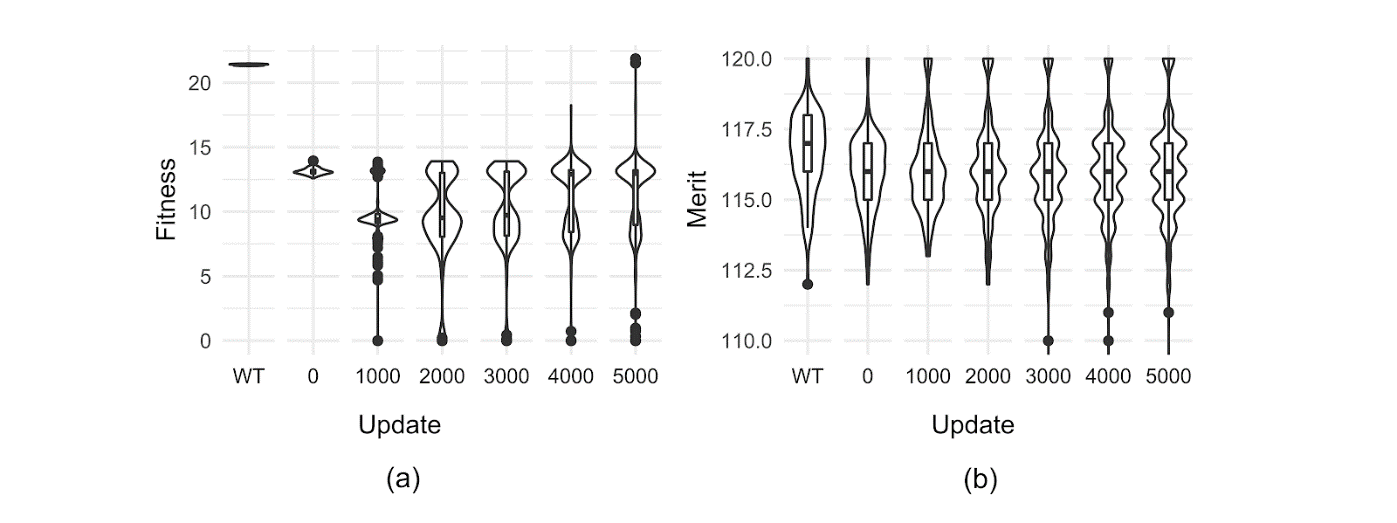


Figure S8. (a) Fitness and (b) merit evolution when recipient genomes sampled from peak L4 are transplanted with peak L0 copy-loop and evolved. Only viable recipient genomes are chosen (Viability ~ 100%). Updates on the x-axis is the evolutionary time for which the hybrid genotypes were evolved. “WT” denotes these measures for the recipient genomes without copy-loop replacement.


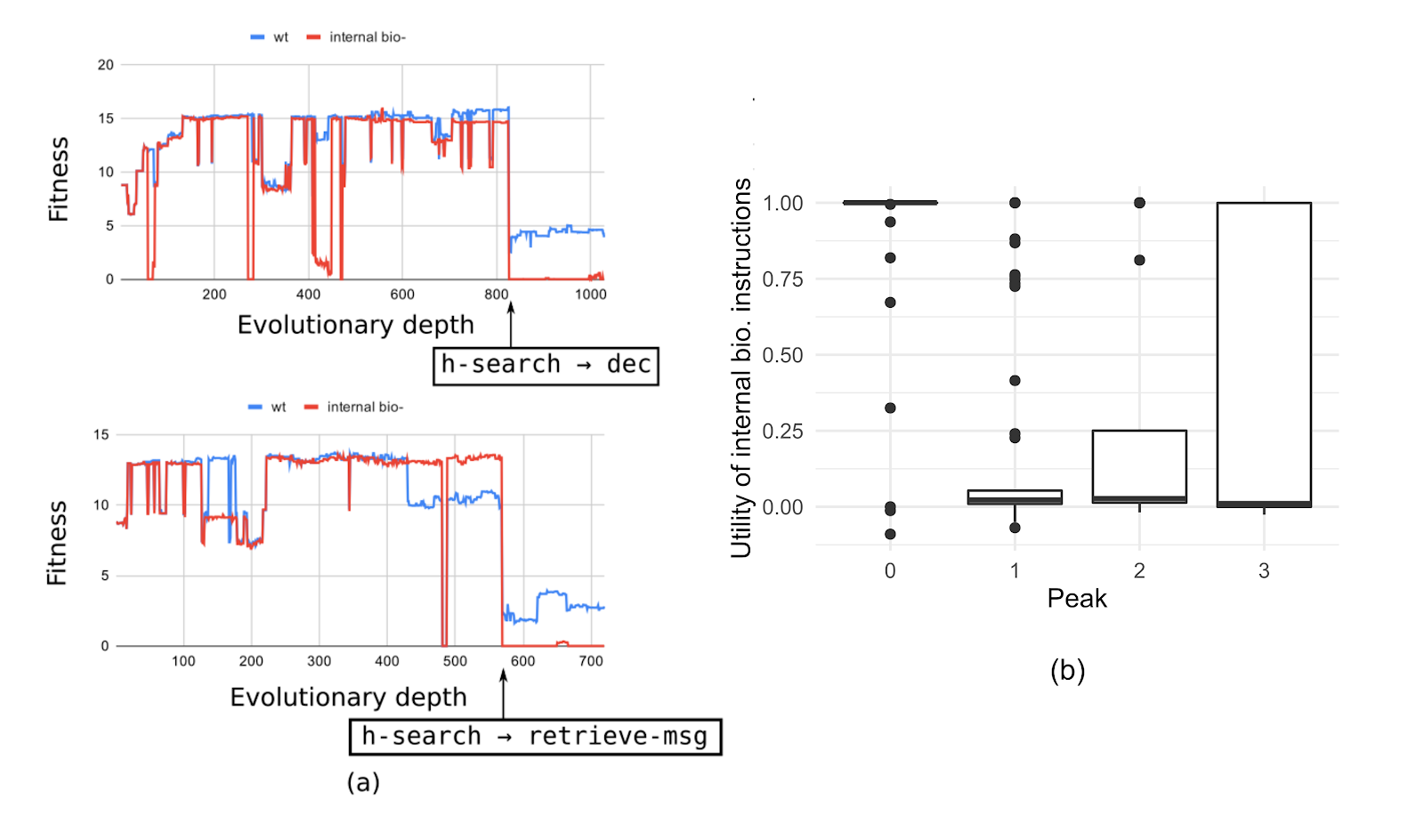


Figure S9. (a) Two representative curves showing the temporal fitness variation of genome lineages from peak L0. The major fitness decline is shown by arrows indicating the mutation that happens at these events. Note that the internal biological instruction knockouts immediately fall to a fitness of zero at these steps. The red line shows the fitness for the lineage after internal biological instructions are knocked out. The difference in the wild type and this fitness is seen only after the loss of h-search indicating the internalization is pre-existent (b) Internal biological instructions generate fitness to a large extent only for genomes from peak L0.


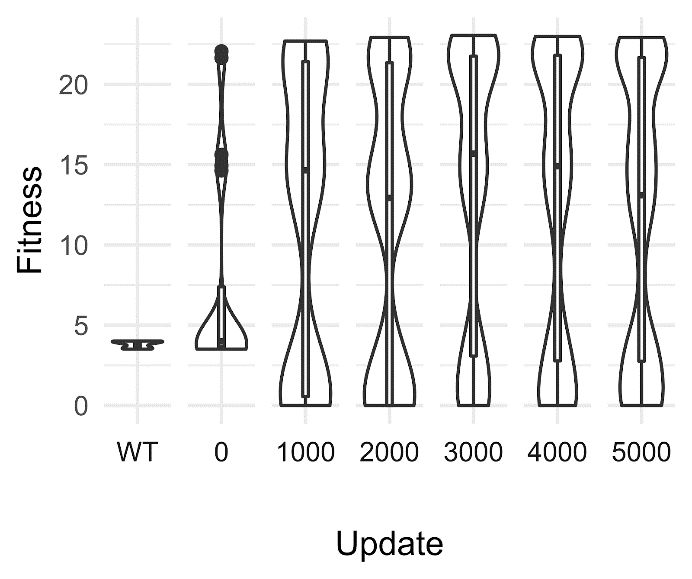


Figure S10. Fitness evolution when recipient genomes sampled from peak H0 are transplanted with H3 copy-loop and evolved. Only viable recipient genomes are chosen (Viability ~ 20%). Updates on the x-axis is the evolutionary time for which the hybrid genotypes were evolved. “WT” denotes these measures for the recipient genomes without copy-loop replacement.


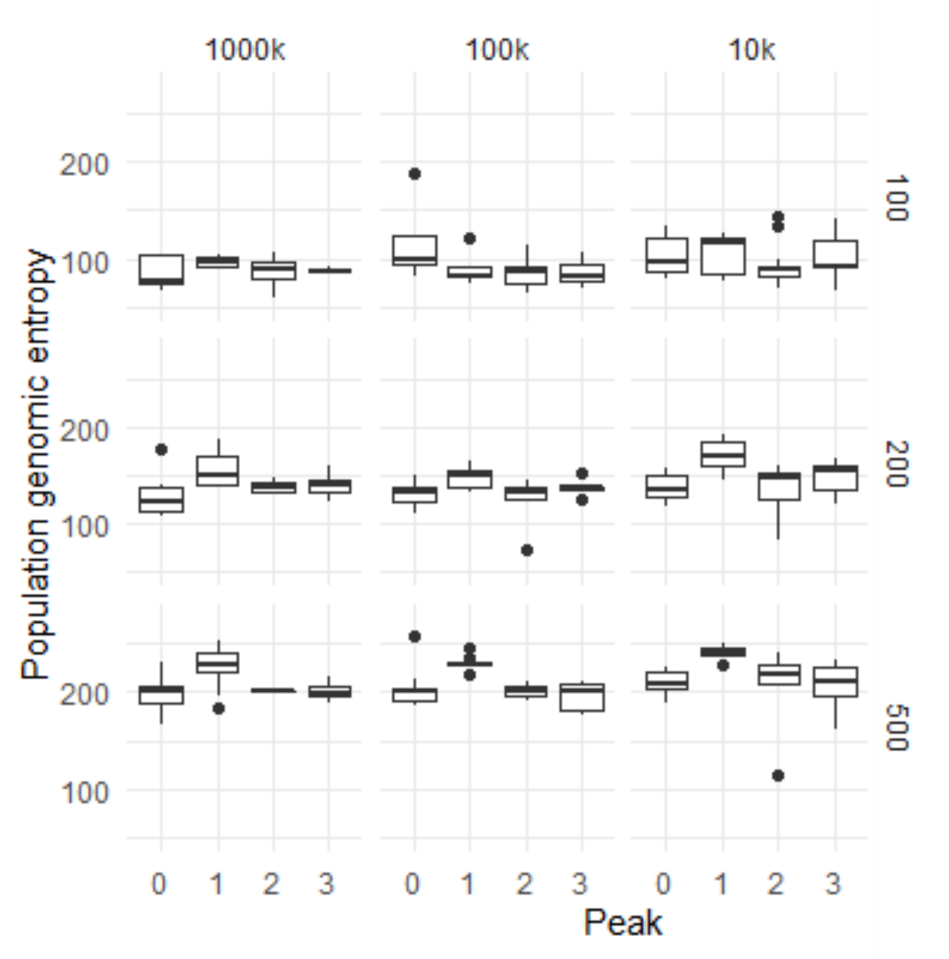


Figure S11. Genomic heterogeneity for populations belonging to the different peaks obtained at a high mutation rate measured by calculating the sum of per-site genomic entropies - divided by resource availability and population size.


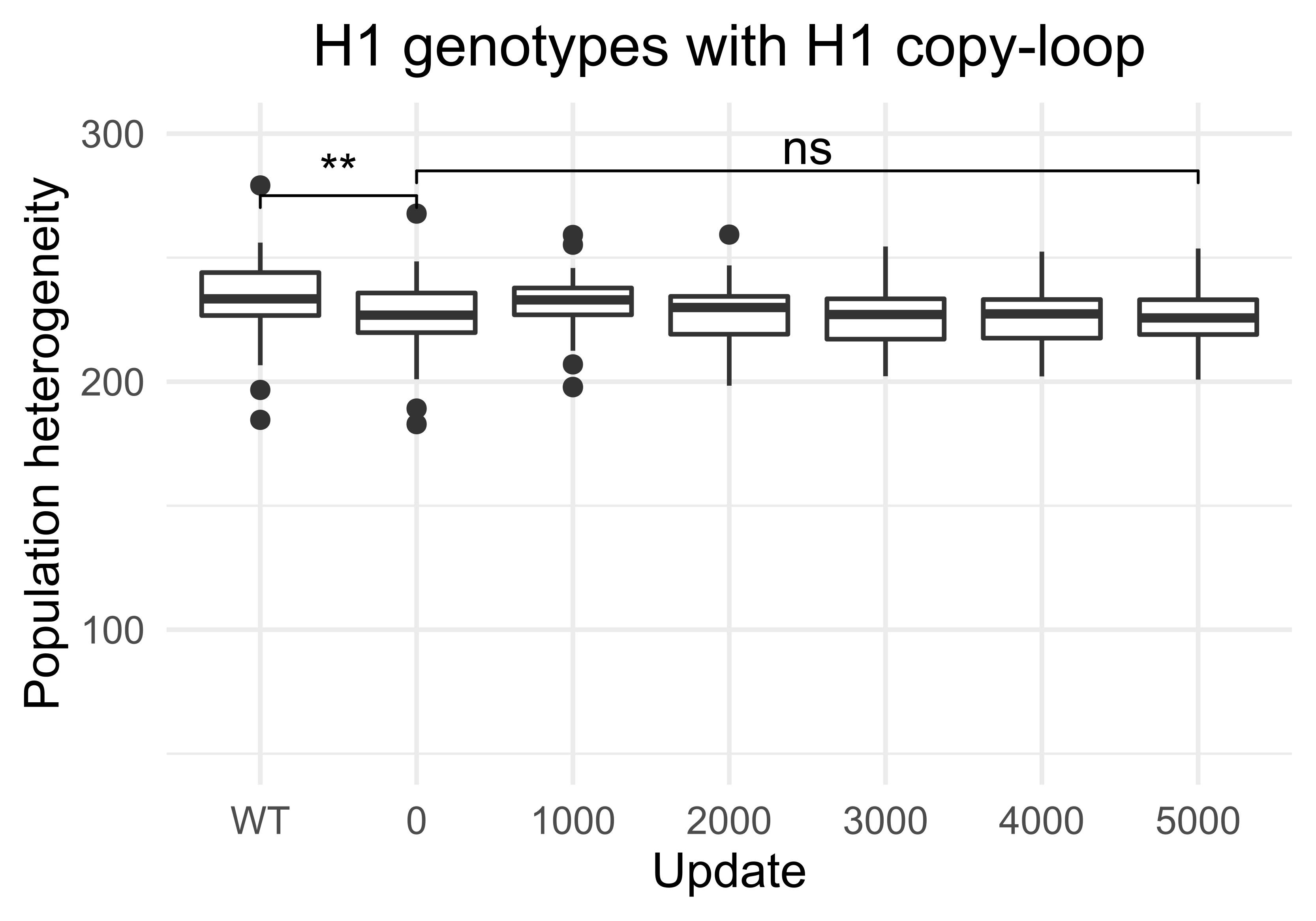


Figure S12. Evolution of population heterogeneity when populations dominated by H1 genotypes are transplanted with a single H1 copy-loop. (Populations evolved under maximum size 500 and 1000k resource availability)


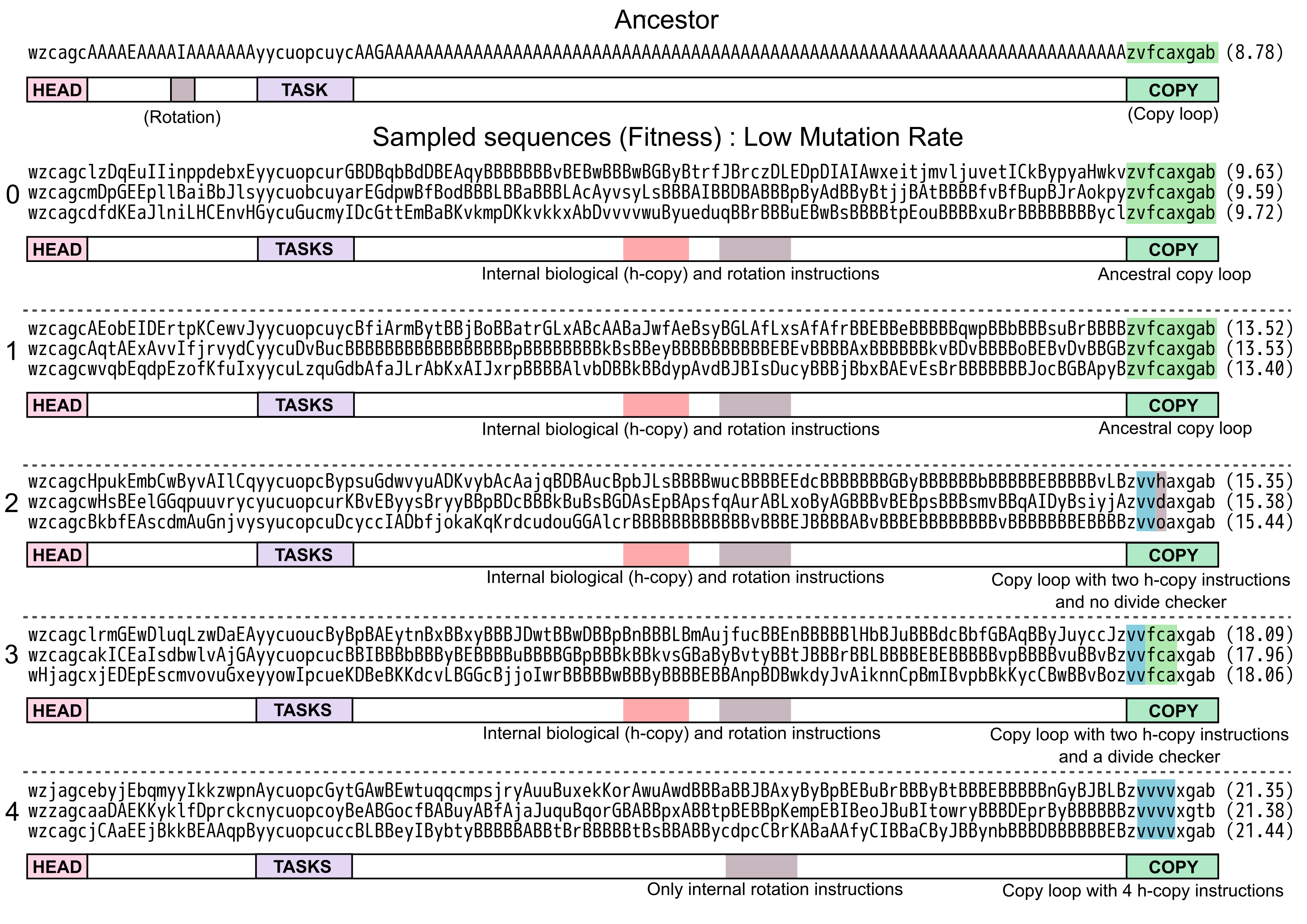


Figure S13. Representative sample sequences (and major sequence differences) from peaks L0-L4 obtained at low mutation rate. Fitness of the genotypes are given in brackets.


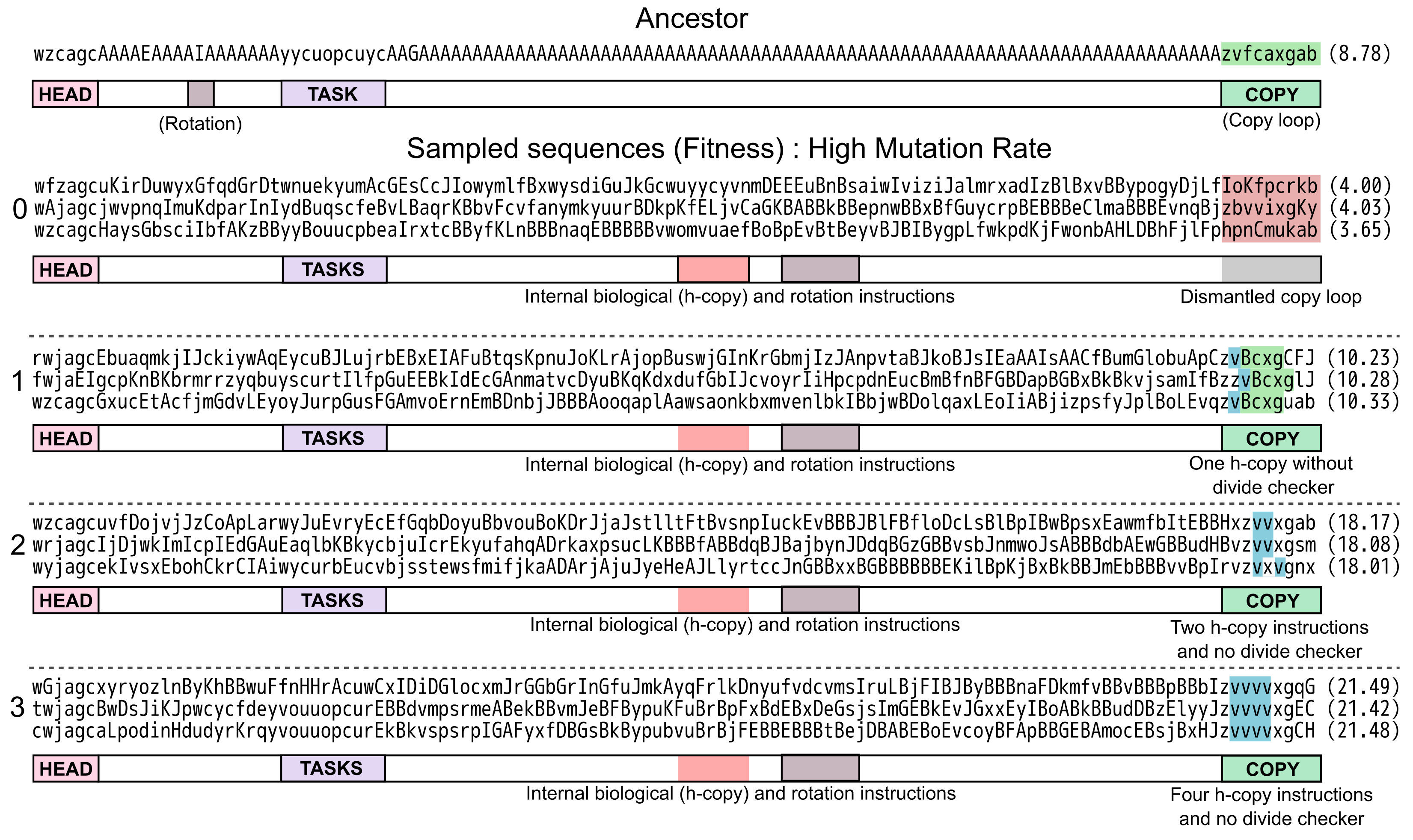


Figure S14. Representative sample sequences (and major sequence differences) from peaks H0-H3 obtained at high mutation rate. Fitness of the genotypes are given in brackets.


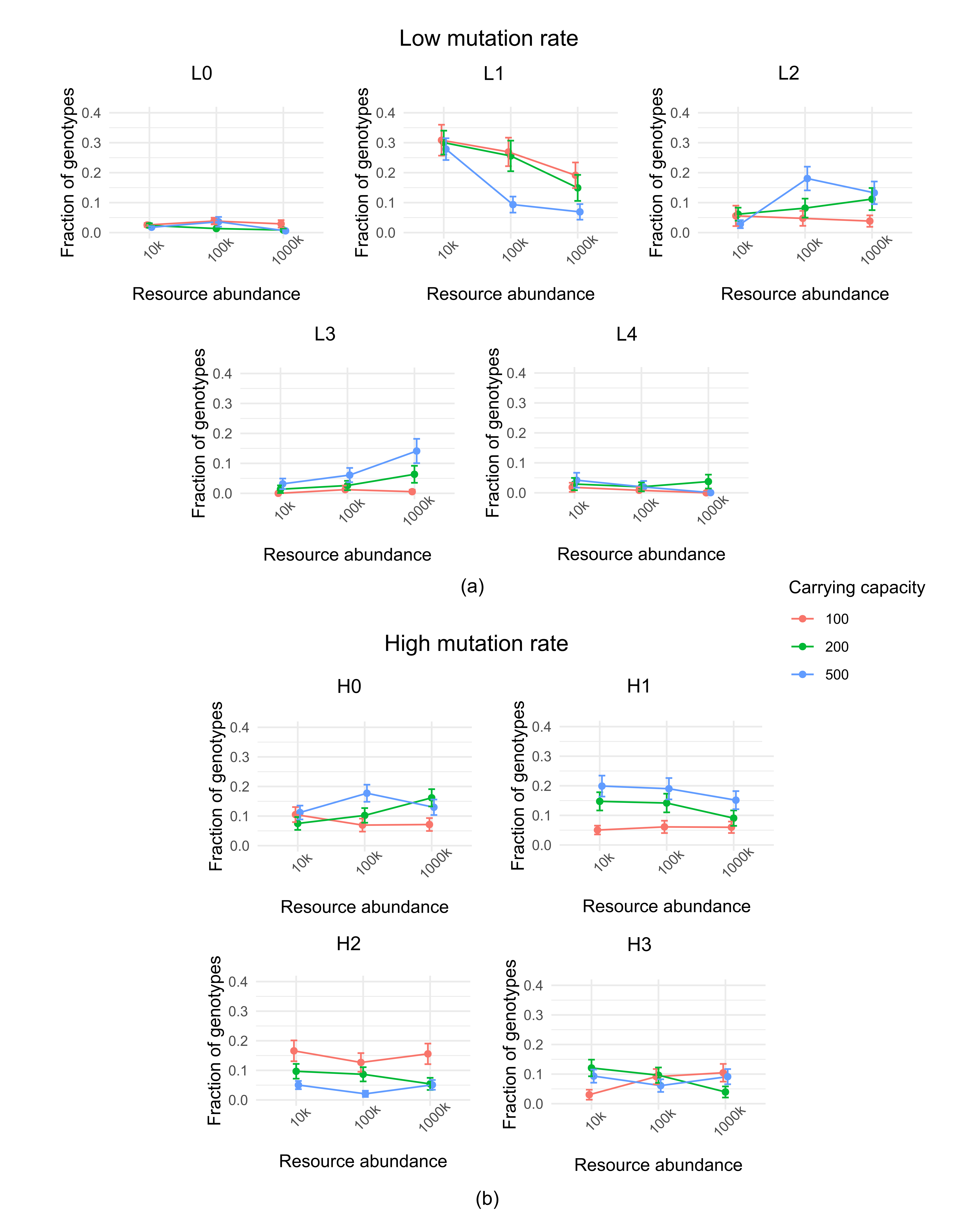


Figure S15. Graphs showing fractional proportion of genotypes of peak L0-L4 (a) and H0-H3 (b) obtained from the fitness spectra from simulations run at low- and high- mutation rates respectively (see Figure 1A and 1B) at 3 resource levels (10k, 100k, and 1000k) with population size limits of 100 (orange), 200 (green) and 500 (orange). (Bootstrap analysis with a sampling size of N=60 out of 100 populations)

| **Logical Task** | **Operation** | **Output (A=10001, B=01011)** |
| --- | --- | --- |
| NOT | ~A | 01110 |
| NAND | ~ (A ∧ B) | 11110 |
| AND | A ∧ B | 00001 |
| ORN | A ∨ ~B, ~A ∨ B | 10101, 01111 |
| OR | A ∨ B | 11011 |
| ANDN | A ∧ ~B, ~A ∧ B | 10000, 01010 |
| NOR | ~ (A ∨ B) | 00100 |
| XOR | (A ∧ ~B) ∨ (~A ∧ B) | 11010 |
| EQU | (A ∧ B) ∨ (~A ∧ ~B) | 00101 |

Table S1. Examples for the nine logical tasks applied to given values of A and B. The symbols used in the table are: “~” denotes logical negation, “∨” denotes logical-or function, and “∧” denotes logical-and function. The logical operations are performed in a bit-wise manner.


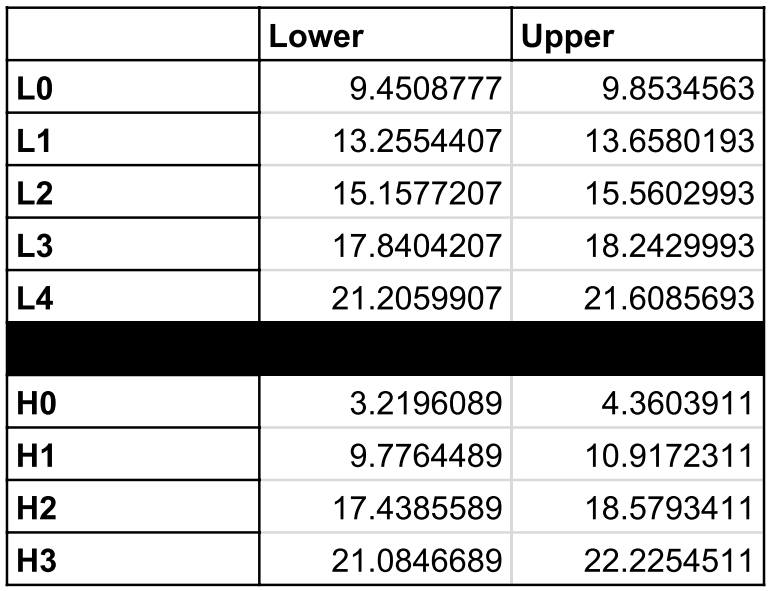


Table S2. Peak fitness ranges used for classification of genotypes. This range is calculated by taking a window equal in size to twice the distribution bandwidth around the maxima obtained from the cumulative fitness distribution.


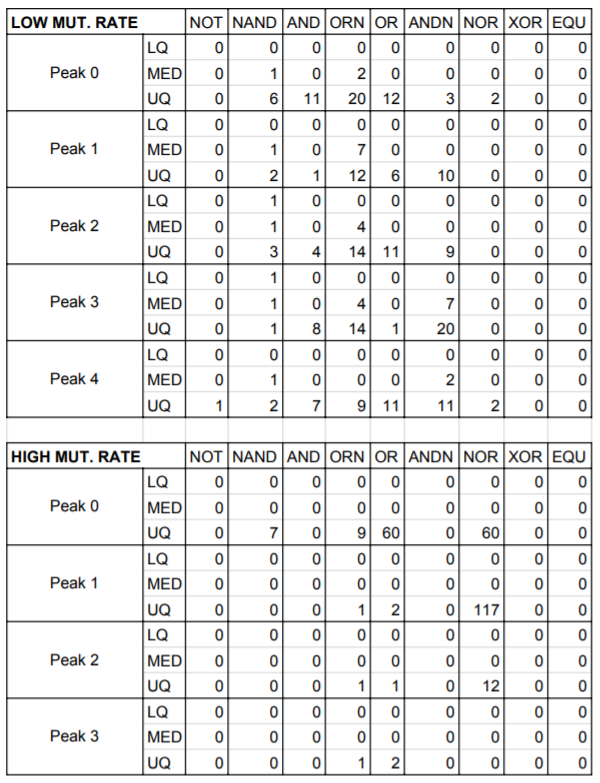


Table S3. Lower quartile (LQ), upper quartile (UQ) and median values of task instances for each task for genomes from low and high mutation rates. (Data for Figure 3c and Figure 4c)
